# Title and abstract screening for literature reviews using large language models: an exploratory study in the biomedical domain

**DOI:** 10.1186/s13643-024-02575-4

**Published:** 2024-06-15

**Authors:** Fabio Dennstädt, Johannes Zink, Paul Martin Putora, Janna Hastings, Nikola Cihoric

**Affiliations:** 1 Department of Radiation Oncology, Cantonal Hospital of St. Gallen, St. Gallen, Switzerland; 2 Institute for Computer Science, University of Würzburg, Würzburg, Germany; 3 Department of Radiation Oncology, Inselspital, Bern University Hospital and University of Bern, Bern, Switzerland; 4 Institute for Implementation Science in Health Care, University of Zurich, Zurich, Switzerland; 5 School of Medicine, University of St. Gallen, St. Gallen, Switzerland; 6 Swiss Institute of Bioinformatics, Lausanne, Switzerland

**Keywords:** Natural language processing, Systematic literature review, Biomedicine, Title and abstract screening, Large language models

## Abstract

**Background:**

Systematically screening published literature to determine the relevant publications to synthesize in a review is a time-consuming and difficult task. Large language models (LLMs) are an emerging technology with promising capabilities for the automation of language-related tasks that may be useful for such a purpose.

**Methods:**

LLMs were used as part of an automated system to evaluate the relevance of publications to a certain topic based on defined criteria and based on the title and abstract of each publication. A Python script was created to generate structured prompts consisting of text strings for instruction, title, abstract, and relevant criteria to be provided to an LLM. The relevance of a publication was evaluated by the LLM on a Likert scale (low relevance to high relevance). By specifying a threshold, different classifiers for inclusion/exclusion of publications could then be defined. The approach was used with four different openly available LLMs on ten published data sets of biomedical literature reviews and on a newly human-created data set for a hypothetical new systematic literature review.

**Results:**

The performance of the classifiers varied depending on the LLM being used and on the data set analyzed. Regarding sensitivity/specificity, the classifiers yielded 94.48%/31.78% for the FlanT5 model, 97.58%/19.12% for the OpenHermes-NeuralChat model, 81.93%/75.19% for the Mixtral model and 97.58%/38.34% for the Platypus 2 model on the ten published data sets. The same classifiers yielded 100% sensitivity at a specificity of 12.58%, 4.54%, 62.47%, and 24.74% on the newly created data set. Changing the standard settings of the approach (minor adaption of instruction prompt and/or changing the range of the Likert scale from 1–5 to 1–10) had a considerable impact on the performance.

**Conclusions:**

LLMs can be used to evaluate the relevance of scientific publications to a certain review topic and classifiers based on such an approach show some promising results. To date, little is known about how well such systems would perform if used prospectively when conducting systematic literature reviews and what further implications this might have. However, it is likely that in the future researchers will increasingly use LLMs for evaluating and classifying scientific publications.

**Supplementary Information:**

The online version contains supplementary material available at 10.1186/s13643-024-02575-4.

## Background

Systematic literature reviews (SLRs) summarize knowledge about a specific topic and are an essential ingredient for evidence-based medicine. Performing an SLR involves a lot of effort, as it requires researchers to identify, filter, and analyze substantial quantities of literature. Typically, the most relevant out of thousands of publications need to be identified for the topic and key information needs to be extracted for the synthesis. Some estimates indicate that systematic reviews typically take several months to complete [1, 2], which is why the latest evidence may not always be taken into consideration.

Title and abstract screening forms a considerable part of the systematic reviewing workload. In this step, which typically follows defining a search strategy and precedes the full-text screening of a smaller number of search results, researchers determine whether a certain publication is relevant for inclusion in the systematic review based on title and abstract. Automating title and abstract screening has the potential to save time and thereby accelerate the translation of evidence into practice. It may also make the reviewing methodology more consistent and reproducible. Thus, the automation or semi-automation of this part of the reviewing workflow has been of longstanding interest [3–5].

Several approaches have been developed that use machine learning (ML) to automate or semi-automate screening [1, 6]. For example, systematic review software applications such as Covidence [7] and EPPI-Reviewer [8] (which use the same algorithm) offer ML-assisted ranking algorithms that aim to show the most relevant publications for the search criteria higher in the reviewing to speed up the manual review process. Elicit [9] is a standalone literature discovery tool that also offers an ML-assisted literature search facility. Furthermore, several dedicated tools have been developed to specifically automate title and abstract screening [1, 10]. Examples include Rayyan [11], DistillerSR [12], Abstrackr [13], RobotAnalyst [14], and ASReview [5]. These tools typically work via different technical strategies drawn from ML and topic modeling to enable the system to learn how similar new articles are to a core set of identified ‘good’ results for the topic. These approaches have been found to lead to a considerable reduction in the time taken to complete systematic reviews [15].

Most of these systems require some sort of pre-selection or specific training for the larger corpus of publications to be analyzed (e.g., identification of some “relevant” publications by a human so that the algorithm can select similar papers) and are thus not fully automated.

Furthermore, dedicated models are required that are built for the specific purpose together with appropriate training data. Fully automated systems that achieve high levels of performance and can be flexibly applied to various topics have not yet been realized.

Large language models (LLMs) are an approach to natural language processing in which very large-scale neural networks are trained on vast amounts of textual data to generate sequences of words in response to input text. These capable models are then subject to different strategies for additional training to improve their performance on a wide range of tasks. Recent technological advancements in model size, architecture, and training strategies have led to general-purpose dialog LLMs achieving and exceeding state-of-the-art performance on many benchmark tasks including medical question answering [16] and text summarization [17].

Recent progress in the development of LLMs led to very capable models. While models developed by private companies such as GPT-3/GPT-3.5/GPT-4 from OpenAI [18] or PaLM and Gemini from Google [19, 20] are among the most powerful LLMs currently available, openly available models are actively being developed by different stakeholders and in some cases achieve performances not far from the state of the art [21].

LLMs have shown remarkable capabilities in a variety of subjects and tasks that would require a profound understanding of text and knowledge for a human to perform. Among others, LLMs can be used for classification [22], information extraction [23], and knowledge access [24]. Furthermore, they can be flexibly adapted via prompt engineering techniques [25] and parameter settings, to behave in a desired way. At the same time, considerable problems with the usage of LLMs such as “hallucinations” of models [26], inherent biases [27, 28], and weak alignment with human evaluation [29] have been described. Therefore, even though the text output generated by LLMs is based on objective statistical calculations, the text output itself is not necessarily factual and correct and furthermore incorporates subjectivity based on the training data. This implies, that an LLM-based evaluation system has a priori some fundamental limitations. However, using LLMs for evaluating scientific publications is a novel and interesting approach that may be helpful in creating fully automated and still flexible systems for screening and evaluating scientific literature.

To investigate whether and how well openly available LLMs can be used for evaluating the relevance of publications as part of an automated title and abstract screening system, we conducted a study to evaluate the performance of such an approach in the biomedical domain with modern openly available LLMs.

## Methods

### Using LLMs for title and abstract screening

We designed an approach for evaluating the relevance of publications based on title and abstract using an LLM. This approach is based on the following strategy:

- An instruction prompt to evaluate the relevance of a scientific publication for inclusion into an SLR is given to an LLM.
- The prompt includes the title and abstract of the publication and the criteria that are considered relevant.
- The prompt furthermore includes the request to return just a number as an answer, which corresponds to the relevance of the publication on a Likert scale (“not relevant” to “highly relevant”).
- The prompt for each publication is created in a structured and automated way.
- A numeric threshold may be defined which separates relevant publications from irrelevant publications (corresponding to the definition of a classifier).

The prompts are created in the following way:

Prompt = [Instruction] + [Title of publication] + [Abstract of publication] + [Relevant Criteria**]**.

(“ + ” is not part of the final prompt but indicates the merge of the text strings).

[Instruction] is the text string describing the general instruction for the LLM to evaluate the publication. The LLM is asked to evaluate the relevance of a publication for an SLR on a numeric scale (low relevance to high relevance) based on the title and abstract of the publication and based on defined relevant criteria.

[Title of publication] is the text string “Title:” together with the title of the publication.

[Abstract of publication] is the text string “, Abstract:” together with the abstract of the publication.

[Relevant Criteria] is the text that describes the criteria to evaluate the relevance of a publication. The relevant criteria are defined beforehand by the researchers depending on the topic to determine which publications are relevant. The [Relevant Criteria] text string remains unchanged for all the publications that should be checked for relevance.

The answer to the LLM usually consists just of a digit on a numeric scale (e.g., 1–5). However, variations are acceptable if the answer can unambiguously be assigned to one of the possible scores on the Likert scale (e.g., the answer “The relevance of the publication is 3.” can unambiguously be assigned to the score 3). This assignment of answers to a score can be automated with a string-search command, meaning a simple regular expression command searching for a positive integer number, which will be extracted from the text string.

A request is sent to the LLM for each publication in the corpus. In cases for which an LLM provided an invalid (unprocessable) response for a publication, that response was excluded from the direct downstream analysis. It was determined for how many publications invalid responses were given and how many of these publications would have been relevant.

**Fig. 1:**
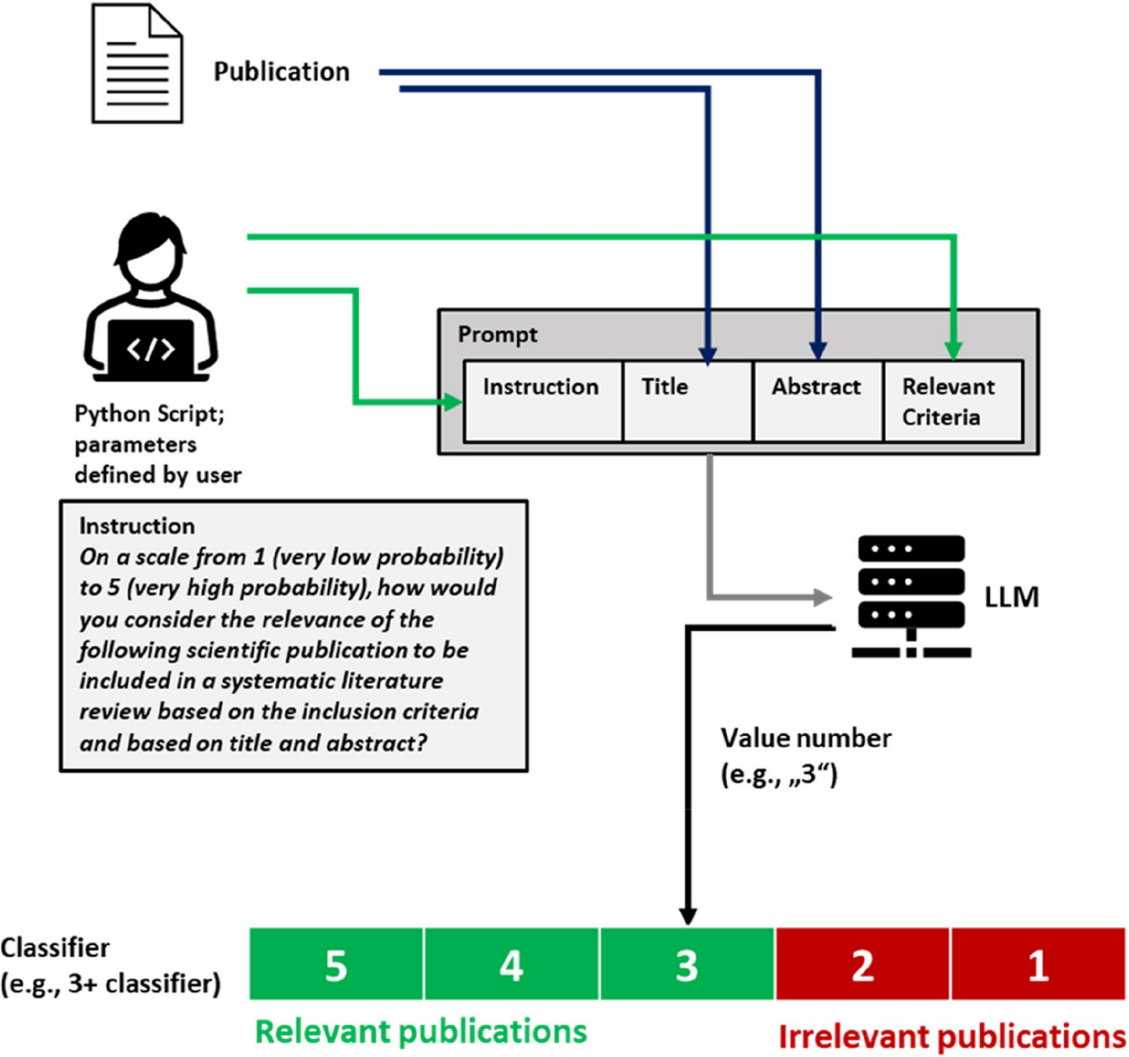
Schematic illustration of the LLM-based approach for evaluating the relevance of a scientific publication. In this example, a 1–5 scale and a 3 + classifier are used

A schematic illustration of the approach is shown in Fig. 1. An example of a prompt is provided in Supplementary material 1: Appendix 1.

A Python script was created to automate the process and to apply it to a data set with a collection of different publications.

With the publications being sorted into different relevance groups, a threshold can be defined, which is used by a classifier to separate relevant from irrelevant publications. For example, a 3 + classifier would classify publications with a score of ≥ 3 as relevant, and publications with a score < 3 as irrelevant.

### Evaluation

The performance of the approach was tested with different LLMs, data sets and settings as described in the following:

### Language models

A variety of different models were tested. To investigate the approach with different LLMs (that are also diverse regarding design and training data), the following four models were used in the experiments:

- FlanT5-XXL (FlanT5) is an LLM developed by Google Research. It’s a variant of the T5 (text-to-text) model, that utilizes a unified text-to-text framework allowing it to perform a wide range of NLP tasks with the same model architecture, loss function, and hyperparameters. FlanT5 is a variant that was enhanced through fine-tuning over a thousand additional tasks and supporting more languages. It is primarily used for research in various areas of natural language processing, such as reasoning and question-answering [30, 31].
- OpenHermes-2.5-neural-chat-7b-v3-1-7B (OHNC) [32] is a powerful open-source LLM, which was merged from the two models OpenHermes 2.5 Mistral 7B [33] and Neural-Chat (neural-chat-7b-v3-1) [34]. Despite having only 7 billion parameters it performs better than some larger models on various benchmarks.
- Mixtral-8 × 7B-Instruct v0.1 (Mixtral) is a pretrained generative Sparse Mixture of Experts LLM developed by Mistral AI [35, 36]. It was reported to outperform powerful models like gpt-3.5-turbo, Claude-2.1, Gemini Pro, and Llama 2 70B-chat on human benchmarks.
- Platypus2-70B-Instruct (Platypus 2) is a powerful language model with 70 Billion parameters [37]. The model itself is a merge of the models Platypus2-70B and SOLAR-0-70b-16bit (previously published as LLaMa-2-70b-instruct-v2) [38].

### Data sets

#### Published data sets

A list of several data sets for SLRs is provided to the public by the research group of the ASReview tool [39]. The list contains data sets on a variety of different biomedical subjects of previously published SLRs. For testing the LLM approach on an individual data set, the [Relevant Criteria] string for each data set was created based on the description in the publication of the corresponding SLR. We tested the approach on a total of ten published data sets covering different biomedical topics (Table 1, Supplementary material 2: Appendix 2).

**Table 1:** Published data sets used for testing the approach

Name	Topic	Number of publications	Relevant publications (%)	Reference
Appenzeller-Herzog_2020	Wilson’s Disease	3479	26 (0.75%)	[40]
Bos_2018	Cerebral small vessel disease and dementia	5756	10 (0.18%)	[41]
Donners_2021	Emicizumab	660	15 (2.27%)	[42]
Jeyaraman_2021	Osteoarthritis	1194	96 (2.26%)	[43]
Leenaars_2020	Rheumatoid arthritis	9543	792 (8.30%)	[44]
Mejboom_2021	TNFα-inhibitors and biosimilars	2224	37 (1.66%)	[45]
Muthu_2021	Spine surgery	3254	354 (10.88%)	[46]
Oud_2018	Borderline personality disorder	1053	20 (1.90%)	[47]
van_de_Schoot_2018	PTSD	6225	38 (0.61%)	[48]
Wolters_2018	Dementia and heart disease	5038	19 (0.38%)	[49]

#### Newly created data set on CDSS in radiation oncology

To test the approach also in a prospective setting on a not previously published review, we created a data set for a new, hypothetical SLR, for which title and abstract screening should be performed.

The use case was an SLR on “Clinical Decision Support System (CDSS) tools for physicians in radiation oncology”. A CDSS is an information technology system developed to support clinical decision-making. This general definition may include diagnostic tools, knowledge bases, prognostic models, or patient decision aids [50]. We decided that the hypothetical SLR should be only about software-based systems to be used by clinicians for decision-making purposes in radiation oncology. We defined the following criteria for the [Relevant Criteria] text of the provided prompt:

- Only inclusion of original articles, exclusion of review articles.
- Publications examining one or several clinical decision-support systems relevant to radiation therapy.
- Decision-support systems are software-based.
- Exclusion of systems intended for support of non-clinicians (e.g., patient decision aids).
- Publications about models (e.g., prognostic models) should only be included if the model is intended to support clinical decision-making as part of a software application, which may resemble a clinical decision support system.

The following query was used for searching relevant publications on PubMed: “(clinical decision support system) AND (radiotherapy OR radiation therapy)”.

Titles and abstracts of all publications found with the query were collected. A human-based title and abstract screening was performed to obtain the ground truth data set. Two researchers (FD and NC) independently labeled the publications as relevant/not relevant based on the title and abstract and based on the [Relevant criteria] string. The task was to label those publications relevant that may be of interest and should be analyzed as full text, while all other publications should be labeled irrelevant. After labeling all publications, some of the publications were deemed relevant only by one of the two researchers. To obtain a final decision, a third researcher (PMP) independently did the labeling for the undecided cases.

The aim was to create a human-based data set purely representing the process of title and abstract screening without further information or analysis.

A manual title and abstract screening was conducted on 521 publications identified in the search with 36 publications being identified as relevant and labeled accordingly in the data set. This data set was named “CDSS_RO”. It should be noted that this data set is qualitatively different from the 10 published data sets, as not only the publications that may be finally included in an SLR are labeled as relevant, but all publications that should be analyzed in full text based on title and abstract. The file is provided at https://github.com/med-data-tools/title-abstract-screening-ai).

### Parameters and settings of LLM-based title and abstract screening

#### Standard parameters

The LLM-based title and abstract screening as described above requires the definition of some parameters. The standard settings for the approach were the following:

- [Instruction] string: We used the following standard [Instruction] string:

“On a scale from 1 (very low probability) to X (very high probability), how would you rate the relevance of the following scientific publication to be included in a systematic literature review based on the relevant criteria and based on title and abstract?”

- Range of scale: defines the range of the Likert scale mentioned in the [Instruction] string (marked as X in the standard string above). For the standard settings, a value of 5 was used.
- Model parameters of the LLMs were defined in the source code. To obtain reproducible results, the model parameters were set accordingly for the model to become deterministic (e.g., the temperature value is a parameter that defines how much variation a response of a model should have. Values greater than 0 add a random element to the output, which should be avoided for the reproducibility of the LLM-based title and abstract screening).

#### Adaptation of instruction prompt and range

The behavior of an LLM is highly dependent on the provided prompt. Adequate adaptation of the prompt may be used to improve the performance of an LLM for certain tasks [25]. To investigate what impact a slightly adapted version of the Instruction prompt would have on the results, we added the string *“(Note: Give a low score if not all criteria are fulfilled. Give only a high score if all or almost all criteria are fulfilled.)”* in the instruction prompt as additional instruction and examined the impact on the performance. Furthermore, the range of the scale was changed from 1–5 to 1–10 in some experiments to investigate what impact this would have on the performance.

### Statistical analyses

The performance of the approach, depending on models and threshold, was determined by calculating the sensitivity (= recall), specificity, accuracy, precision, and F1-score of the system, based on the amount of correctly and incorrectly included/excluded publications for each data set.

### Comparison with the automated classifier of Natukunda et al.

The LLM-based title and abstract screening was compared to another, recently published approach for fully automated title and abstract screening. This approach, developed by Natukunda et al., uses an unsupervised Latent Dirichlet Allocation-based topic model for screening [51]. Unlike the LLM-based approach, it does not require an additional [Relevant Criteria] string, but defined search keywords to determine which publications are relevant. The approach was used to do a screening on the ten published data sets as well as on the CDSS_RO data set. To obtain the required keywords we processed the text of the used search terms by splitting combined text into individual words and removing stop words, duplicates, and punctuation (as described in the original publication of Natukunda et al.).

## Results

### Performance of LLM-based title and abstract screening of different models on published data sets

**Fig. 2:**
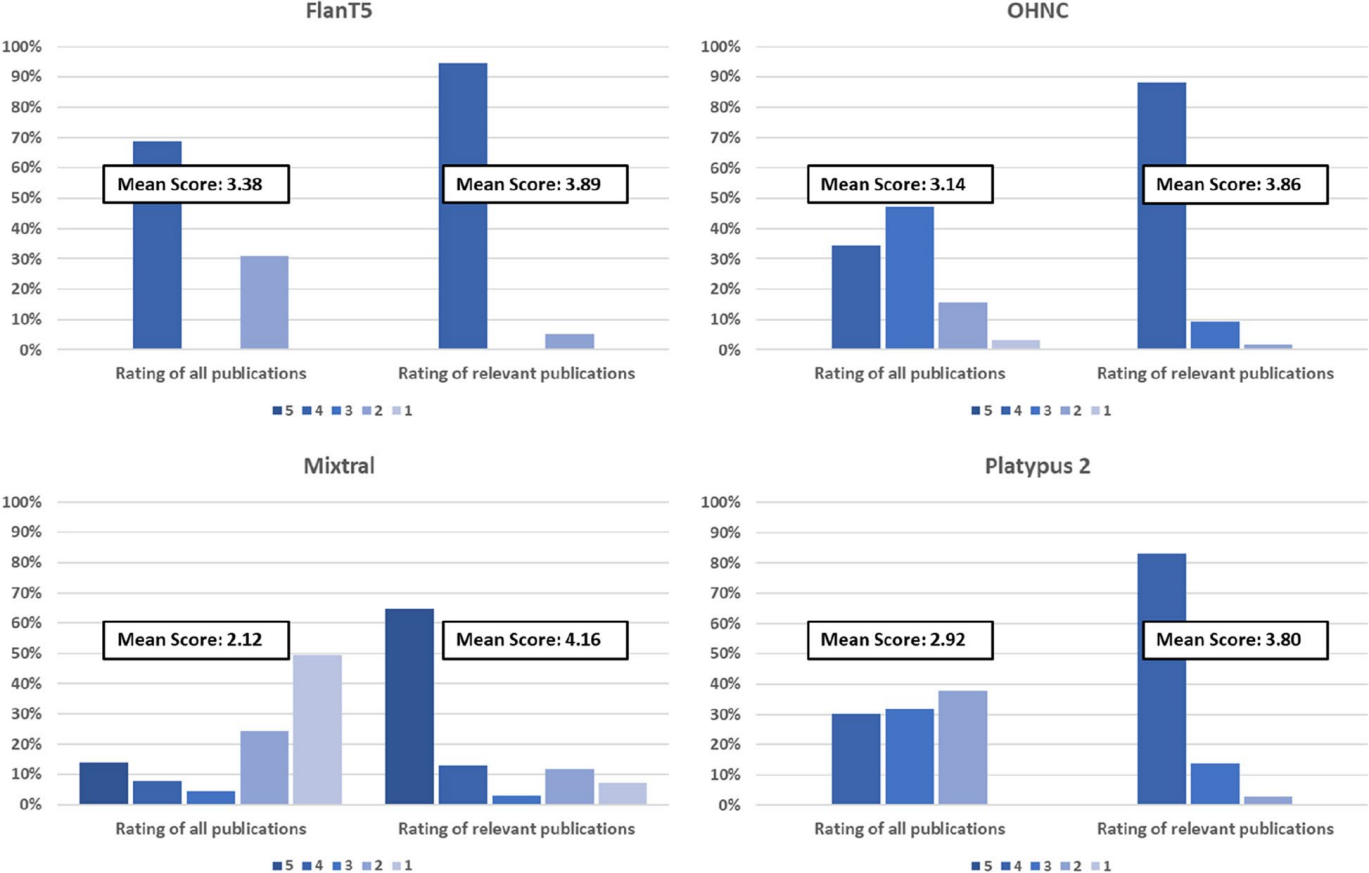
Distribution of scores given by the different models

The LLM-based screening with a Likert scale of 1–5 provided clear results for evaluating the relevance of a publication in the majority of cases. Out of the total of 44,055 publications among the 10 published data sets, valid and unambiguously assignable answers were given for 44,055 publications (100%) by the FlanT5 model, for 44,052 publications (99.993%) by the OHNC model, for 44,026 publications (99.93%) by the Mixtral model and for 44,054 publications (99.998%) by the Platypus 2 model. The few publications for which an invalid answer was given were excluded from further analysis. None of the excluded publications was relevant. The distribution of scores given was different between the different models. For example, the OHNC model ranked the majority of publications with a score of 3 (47.2%) or 4 (34.2%), while the FlanT5 model ranked almost all publications with a score of either 4 (68.1%) or 2 (31.7%). For all models, the group of publications labeled as relevant in the data sets was ranked with higher scores compared to the overall group of publications (mean score of 3.89 compared to 3.38 for FlanT5, 3.86 compared to 3.14 for OHNC, 4.16 compared to 2.12 for Mixtral and 3.80 compared to 2.92 for Platypus 2). An overview is provided in Fig. 2.

Based on the scores given, according classifiers that label publications with a score of greater than or equal to “X” as relevant, have higher rates of sensitivity and lower rates of specificity with decreasing threshold (decreasing “X”).

**Fig. 3:**
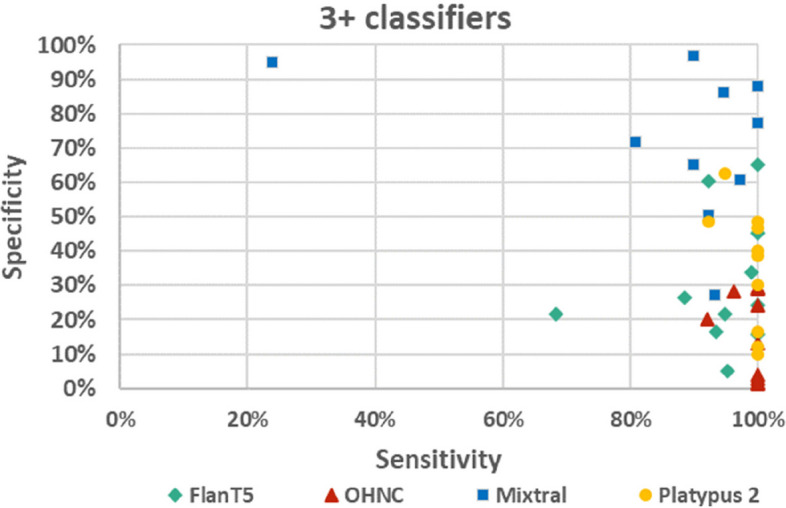
Sensitivity and specificity of the 3 + classifiers on different data sets using different models. Each data point represents the results of one of the data sets

Classifiers with a threshold of ≥ 3 (3 + classifiers) were further analyzed, as these classifiers were considered to correctly identify the vast majority of relevant publications (high sensitivity) without including too many irrelevant publications (sufficient specificity). The 3 + classifiers had a sensitivity/specificity of 94.8%/31.8% for the FlanT5 model, of 97.6%/19.1% for the OHNC model, of 81.9%/75.2% for the Mixtral model, and of 97.2%/38.3% for the Platypus 2 model on all ten published data sets. The performance of the classifiers was quite different depending on the data set used (Fig. 3). Detailed results on the individual data sets are presented in Supplementary material 3: Appendix 3.

The highest specificity at 100% sensitivity was seen for the Mixtral model on the data set Wolters_2018 with all 19 relevant publications being scored with 3–5, while 4410 of 5019 irrelevant publications were scored with 1 or 2 (specificity of 87.87%). The lowest sensitivity was observed with the Mixtral model on the dataset Jeyaraman_2021 with 23.96% sensitivity at 94.63% specificity.

### Using LLM-based title and abstract screening for a new systematic literature review

**Fig. 4:**
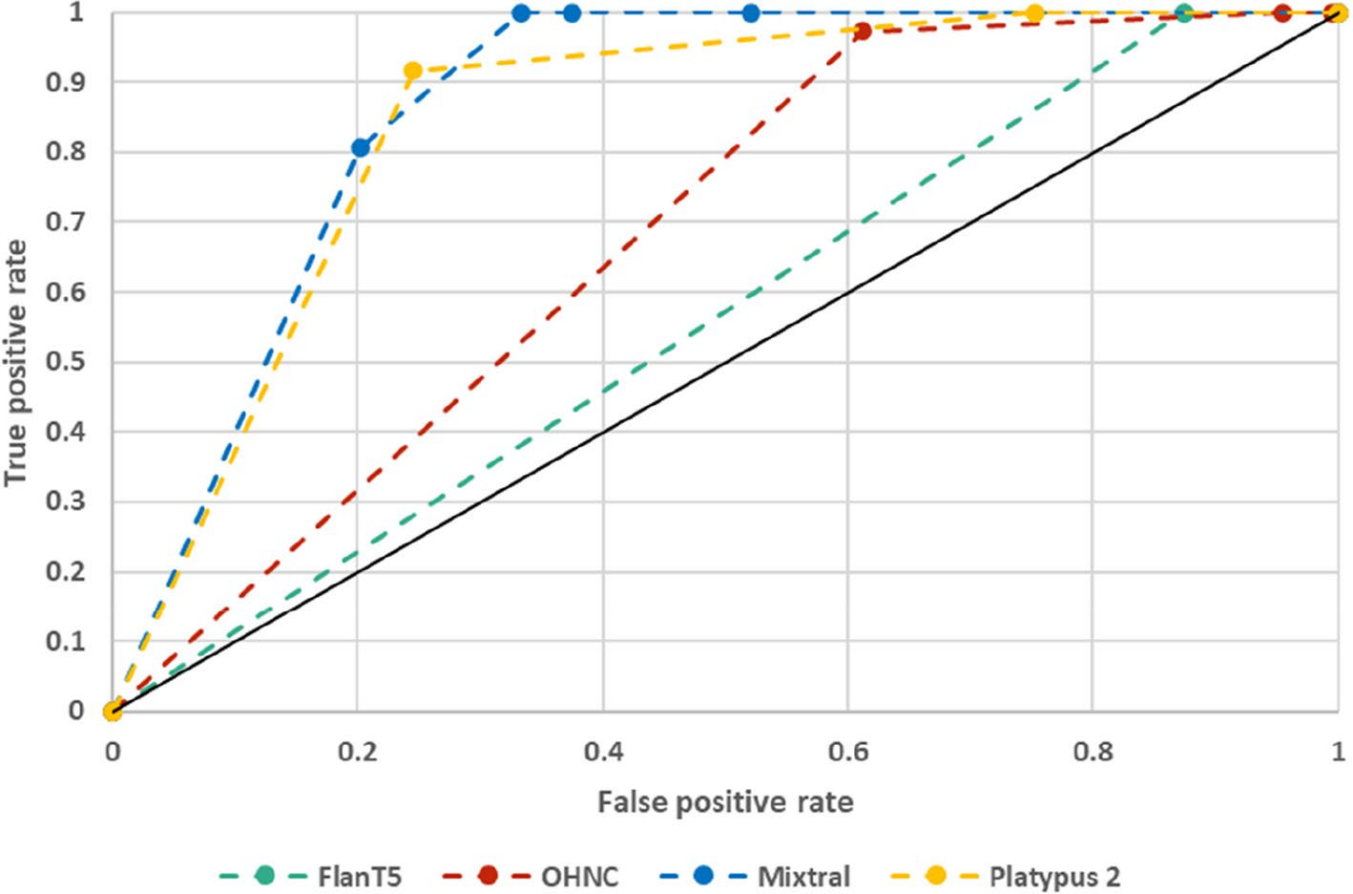
Receiver operating characteristics (ROC) curves of the LLM-based title and abstract screening for the different models on the CDSS_RO data set

On the newly created manually labeled data set, the 3 + classifiers had 100% sensitivity for all four models with specificity ranging from 4.54 to 62.47%. The results of the LLM-based title and abstract screening, dependent on the threshold for the classifiers are presented as receiver operating characteristics (ROC) curves in Fig. 4 as well as in Supplementary material 3: Appendix 3.

### Dependence of LLM-based title and abstract screening on Instruction prompt and on a range of scale

Several runs of the Python script with different settings (adapted [Instruction] string and/or range of scale 1–10 instead of 1–5) were performed, which led to different results. Minor adaptation of the Instruction string with an additional demand to focus on the mentioned criteria had a different impact on the performance of the classifiers depending on the LLM used. While the sensitivity of the 3 + classifiers remained at 100% for all four models, the specificity was lower for the OHNC model (2.89% vs. 4.54%), the Mixtral model (56.29% vs. 62.47%) and the Platypus 2 model (15.88% vs. 24.74%), while it was higher for the FlanT5 model (25.15% vs. 12.58%).

**Fig. 5:**
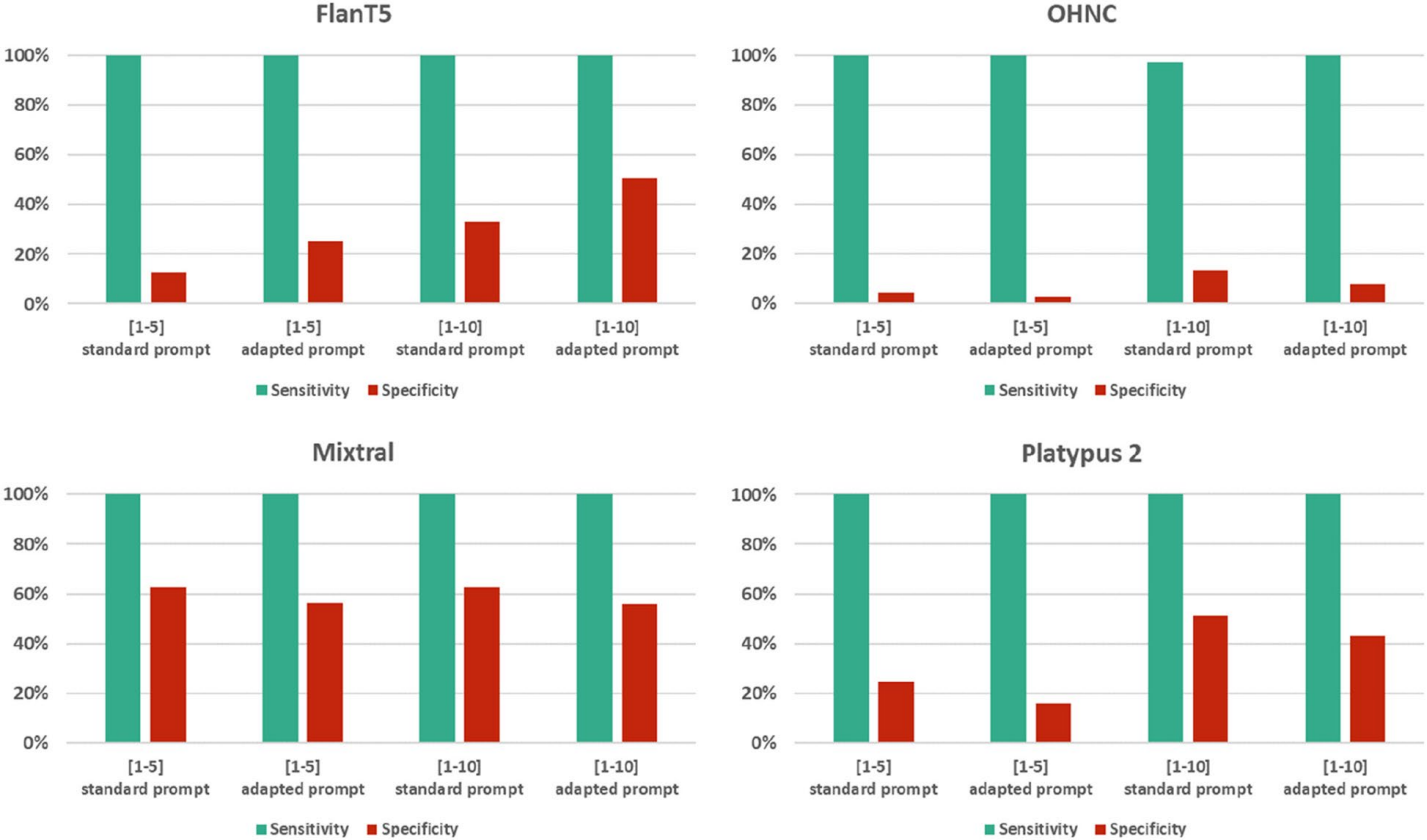
Performance of the classifiers depending on adaptation of the prompt and on the range of scale

Changing the range of scale from 1–5 to 1–10 and using a 6 + classifier instead of a 3 + classifier led to a lower sensitivity for the OHNC model (97.22% vs. 100%), while increasing the specificity (13.49% vs. 4.54%). For the other models, the sensitivity remained at 100% with higher specificity for the Platypus 2 model (51.34% vs. 24.74%) and the FlanT5 model (50.52% vs. 12.58%). The specificity was unchanged for the Mixtral model at 62.47%, which was the highest value among all combinations at 100% sensitivity. No combination of the settings for a range of scales and with/without prompt adaptation was superior among all models. An overview of the results is provided in Fig. 5.

### Comparison with unsupervised title and abstract screening of Natukunda et al.

The screening approach developed by Natukunda et al. achieved an overall sensitivity of 52.75% at 56.39% specificity on the ten published data sets. As for the LLM-based screening, the performance of this approach was dependent on the data set analyzed. The lowest sensitivity was observed for the Jeyaraman_2021 data set (1.04%), while the highest sensitivity was observed for the Wolters_2018 dataset (100%). Compared to the 3 + classifier with the Mixtral model, the LLM-based approach had higher sensitivity on 9 data sets and equal sensitivity on 1 data set, while it had higher specificity on 6 data sets and lower specificity on 4 data sets.

On the CDSS_RO data set, the approach of Natukunda et al. achieved 94.44% sensitivity (lower than all four LLMs) at 39.59% specificity (lower than the Mixtral model and higher than the FlanT5, OHNC, and Platypus 2 models). Further data on the comparison is provided in Supplementary material 4: Appendix 4.

## Discussion

We developed and elaborated a flexible approach to use LLMs for automated title and abstract screening that has shown some promising results on a variety of biomedical topics. Such an approach could potentially be used to automatically pre-screen the relevance of publications based on title and abstract. While the results are far from perfect, using LLMs for evaluating the relevance of publications could potentially be helpful (e.g., as a pre-processing step) when performing an SLR. Furthermore, the approach is widely applicable without the development of custom tools or training custom models.

### Automated and semi-automated screening

A variety of different ML and AI tools have been developed to assist researchers in performing SLRs [5, 10, 52, 53]. Fully automated systems (like the LLM-based approach presented in our study) still fail to differentiate relevant from irrelevant publications near the level of human evaluation [51, 54].

A well-functioning fully automated title and abstract screening system that could be used on different subjects in the biomedical domain and possibly also in other scientific areas would be very valuable. While human-based screening is the current gold standard, it has considerable drawbacks. From a methodological point of view, one major problem of human-based literature evaluation, including title and abstract screening, is the subjectivity of the process [55]. Evaluating the publications (based on title and abstract) is dependent on the experience and individual judgments of the person doing the screening. To overcome this issue, SLRs of high quality require multiple independent researchers to do the evaluation with specific criteria upon inclusion/exclusion defined beforehand [56]. Nevertheless, subjectivity remains an unresolved issue, which also limits the reproducibility of results. From a practical point of view, another major problem is the considerable workload needed to be performed by humans, especially if thousands of publications need to be assessed, which is multiplied by the need to have multiple reviewers and to discuss disagreements. The challenge of workload is not just a matter of inconvenience, as SLRs on subjects that require tens of thousands of publications to be searched, may just not be feasible for small research teams to do, or may already be outdated after the time it would take to do the screening and analyze the results.

While fully automated screening approaches may also be affected by subjectivity (since the training data of models is itself generated by processes which are affected by subjectivity), the results would at least be more reproducible, and automation can be applied at scale in order to overcome the problem of practicability.

While current fully automated systems cannot replace humans in title and abstract screening, they may nevertheless be helpful. Such systems are already being used in systematic reviews and most likely their usage will continue to grow [57].

Ideally, a fully automated system should not miss a single relevant publication (100% sensitivity) while minimizing as far as possible the number of irrelevant publications included. This would allow confident exclusion of some of the retrieved search results which is a big asset to reducing time taken in manual screening.

### LLMs for title and abstract screening

By creating structured prompts with clear instructions, an LLM can feasibly be used for evaluating the relevance of a scientific publication. In comparison to some other solutions, the LLM-based screening may have some advantages. On the one hand, the flexible nature of the approach allows adaptation to a specific subject. Depending on the question, different prompts for relevant criteria and instructions can be used to address the individual research question. On the other hand, the approach can create reproducible results, given a fixed model, parameters, prompting strategy, and defined threshold. At the same time, it is scalable to process large numbers of publications. As we have seen, such an approach is feasible with a performance similar to or even better in comparison to other current solutions like the approach of Natukunda et al. However, it should be noted that the performance varied considerably depending on which of the 10 + 1 data sets were used.

### Further applications of LLMs in literature analysis

While we investigated LLMs for evaluating the relevance of publications and in particular for title and abstract screening, it is being discussed how these models may be used for a variety of tasks in literature analysis [58, 59]. For example, Wang et al. obtained promising results when investigating if ChatGPT may be used for writing Boolean Queries for SLRs [60]. Aydin et al., also using ChatGPT, employed the LLM to write an entire Literature Review about Digital Twins in Healthcare [61].

Guo et al. recently performed a study using the OpenAI API with gpt-3.5 and gpt-4 to create a classifier for clinical reviews [62]. They observed promising results when comparing the performance of the classifier against human-based screening with a sensitivity of 76% at 91% specificity on six different review papers. In contrast to our approach, they used a Boolean classifier instead of a Likert scale. Another approach was developed by Akinseloyin et al., who used ChatGPT to create a method for citation screening by ranking the relevance of publications using a question-answering framework [63].

The question may arise what the purpose of using a Likert scale instead of a direct binary classifier is (also since some models only rarely use some of the score values; see e.g., FlanT5 in Fig. 2). The rationale for using the Likert scale arose out of some preliminary, unsystematic explorations we conducted using different models and ranges of scale (including binary). We realized that using a Likert scale has some advantages as it sorts the publications into several groups depending on the estimated relevance. This also allows flexible adjustment of the threshold (which may potentially also be useful if the user wants to rather focus on sensitivity or rather on specificity).

However, there seem to be several feasible approaches and frameworks to use LLMs for the screening of publications.

It should be noted that an LLM-based approach for evaluating the relevance of publications might just as well be used for a variety of different classification tasks in literature analysis. For example, one may adopt the [Instruction prompt] asking the LLM not to evaluate the relevance of a publication on a Likert scale, but for classification into several groups like “original article”, “trial”, “letter to the editor”, etc. From this point of view, the title and abstract screening is just a special use case of LLM-based classification.

### Future developments

The capabilities of LLMs and other AI models will continue to evolve, which will increase the performance of fully automated systems. As we have seen, the results are highly dependent on the LLM used for the approach. In any case, there may still be substantial room for improvement and optimization and it currently is unclear what LLM-based approach with which prompts, models, and settings yields the best results over a large variety of data sets.

Furthermore, LLMs may not only be used for the screening of titles and abstracts but for the analysis of full-text documents. The newest generation of language and multimodal models may process whole articles or potentially also image data from publications [64, 65]. Beyond that, LLM-based evaluation of scientific data and publications may only be one of several options for AI assistance in literature analysis. Future systems may combine different ML and AI approaches for optimal automated processing of literature and scientific data.

### Limitations of LLM-based title and abstract screening

Even though the LLM-based screening presented in our work shows some promising results, it also has some drawbacks and limitations. While the open framework with adaptable prompts makes the approach flexible, the performance of the approach is highly dependent on the used model, the input parameters/settings, and the data set analyzed. If a slightly different instruction or another scale (1–10 instead of 1–5) is used, this can have a considerable impact on the performance. The classifiers analyzed in our study failed to consistently identify relevant publications at 100% sensitivity without considerably impairing the specificity. In academic research, the bar for automated screening tools needs to be very high, as ideally not a single relevant publication should be missed. The LLM-based title and abstract screening requires the definition of clear criteria for inclusion/exclusion. For research questions with less clear relevance criteria, LLMs may not be that useful for the evaluation. This may potentially be one reason, why the performance of the approach was quite different in our study depending on the data set analyzed. Overall, there are still many open questions, and it is unclear if and how high levels of performance can be consistently guaranteed so that such a system can be relied on. It is interesting that the Mixtral model, even though it seemed to have the highest level of performance on average, performed poorly with low sensitivity on one data set (Fig. 3). Further research is needed to investigate the requirements for good performance of the LLMs in evaluating scientific literature.

Another limitation of the approach in its current form is a considerable demand for resources regarding calculation power and hardware equipment. Answering thousands of long text prompts with modern, multi-billion-parameter LLMs requires sufficient IT infrastructure and calculation power to perform. The issue of resource demand is especially relevant if many thousand publications are evaluated and if very complex models are used.

### Fundamental issues of using LLMs in literature analysis

On a more fundamental level, there are some general issues regarding the use of LLMs for literature studies. LLMs calculate the probability for a sequence of words based on their training data which derives from past observations and knowledge. They can thereby inherit unwanted features and biases (such as for example ethnic or gender biases) [29, 66]. In a recent study by Koo et al., it was shown that the cognitive biases and preferences of LLMs are not the same as the ones of humans as a low correlation between ratings given by LLMs and humans was observed [67]. The authors therefore stated that LLMs are currently not suitable as fair and reliable automatic evaluators. Considering that using LLMs for evaluating and processing scientific publications may be seen as a problematic and questionable undertaking. However, the biases present in language models affect different tasks differently, and it remains to be seen how they might differentially affect different screening tasks in the literature review [28].

Nevertheless, it is most likely that LLMs and other AI solutions will be increasingly used in conducting and evaluating scientific research [68]. While this certainly will provide a lot of chances and opportunities, it is also potentially concerning. The amount and proportion of text being written by AI models is increasing. This includes not only public text on the Internet but also scientific literature and publications [69, 70]. The fact that ChatGPT has been chosen as one of the top researchers of the year 2023 by Nature and has frequently been listed as co-author, shows how immediate the impact of the development has already been [71]. At the same time, most LLMs are trained on large amounts of text provided on the Internet. The idea that in the future LLMs might be used to evaluate publications written with the help of LLMs that may themselves be trained on data created by LLMs may lead to disturbing negative feedback loops which decrease the quality of the results over time [72]. Such a development could actually undermine academia and evidence-based science [73], also due to the known fact that LLMs tend to “hallucinate”, meaning that a model may generate text with illusory statements not based on correct data [26]. It is important to be aware that LLMs are not directly coupled to evidence and that there is no restriction preventing a model from generating incorrect statements. As part of a screening tool assigning just a score value to the relevance of a publication, this may be a mere factor impairing the performance of the system – yet for LLM-based analysis in general this is a major problem.

The majority of studies that so far have been published on using LLMs for publication screening used the currently most powerful models that are operated by private companies—most notably the ChatGPT models GPT-3.5 and GPT-4 developed by OpenAI [18, 74]. Using models that are owned and controlled by private companies and that may change over time is associated with additional major problems when using them for publication screening, such as a lack of reproducibility. Therefore, after initial experiments with such models, we decided to use openly available models for our study.

### Limitations of the study

Our study has some limitations. While we present a strategy for using LLMs to evaluate the relevance of publications for an SLR, our work does not provide a comprehensive analysis of all possible capabilities and limitations. Even though we achieved promising results on ten published data sets and a newly created one in our study, generalization of the results may be limited as it is not clear how the approach would perform on many other subjects within the biomedical domain more broadly and within other domains. To get a more comprehensive understanding, thorough testing with many more data sets about different topics would be needed, which is beyond the scope of this work. Testing the screening approach on retrospective data sets is also per se problematic. While a good performance on retrospective data should hopefully indicate a good performance if used prospectively on a new topic, this does not have to be the case [75]. Indeed, naively assuming a classifier that was tested on retrospective data will perform equally on a new research question is clearly problematic, since a new research question in science is by definition new and unfamiliar and therefore will not be represented in previously tested data sets.

Furthermore, models that are trained on vast amounts of scientific literature may even have been trained on some publications or the reviews that are used in the retrospective benchmarking of an LLM-based classifier, which obviously creates a considerable bias. To objectively assess how well an LLM-based solution can evaluate scientific publications for new research questions, large cultivated and independent prospective data sets on many different topics would be needed, which will be very challenging to create. It is interesting that the LLM-based title and abstract screening in our study would have also performed well on our new hypothetical SLR on CDSS in radiation therapy, but of course, this alone is a too limited data basis from which to draw general conclusions. Therefore, it currently cannot be reliably known in which situations such an LLM-based evaluation may succeed or may fail.

Regarding the ten published data sets, the results also need to be interpreted with caution. These data sets may not truly represent the singular task of title and abstract screening. For example, in the Appenzeller-Herzog_2020 data set, only the 26 publications that were finally included (not only after title and abstract screening but also after further analysis) were labeled as relevant [40]. While these publications ideally should be correctly identified by an AI-classifier, there may be other publications in the data set, that per se cannot be excluded solely based on title and abstract. Furthermore, we had to retrospectively define the [Relevant Criteria] string based on the text in the publication of the SLR. This obviously is a suboptimal way to define inclusion and exclusion criteria, as the defined string may not completely align with the criteria intended by the researchers of the SLR.

We also want to emphasize that the comparison with the approach of Natukunda et al. needs to be interpreted with caution since the two approaches are not based on exactly the same prerequisites: the LLM-based approach requires a [Relevant Criteria] string, while the approach of Natukunda et al. requires defined keywords.

While overall our work shows that LLM-based title and abstract screening is possible and shows some promising results on the analyzed data sets, our study cannot fully answer the question of how well LLMs would perform if they were used for new research. Even more importantly, we cannot answer the question of to what extent LLMs should be used for conducting literature reviews and for doing research.

## Conclusions

Large language models can be used for evaluating the relevance of publications for SLRs. We were able to implement a flexible and cross-domain system with promising results on different biomedical subjects. With the continuing progress in the fields of LLMs and AI, fully automated computer systems may assist researchers in performing SLRs and other forms of scientific knowledge synthesis. However, it remains unclear how well such systems will perform when being used in a prospective manner and what implications this will have on the conduction of SLRs.

### Supplementary Information

Supplementary Material 1: Appendix 1: Sample prompt.

Supplementary Material 2: Appendix 2: Relevant criteria of published datasets.

Supplementary Material 3: Appendix 3: Performance of models on data sets.

Supplementary Material 4: Appendix 4: Comparison with other approach.

## Data Availability

All data generated and analyzed during this study are either included in this published article (and its supplementary information files) or publicly available on the Internet. The Python script as well as the CDSS_RO data set are available under https://github.com/med-data-tools/title-abstract-screening-ai. The ten published data sets analyzed in our study are available on the GitHub Repository of the research group of the ASReview Tool [39].

## Abbreviations

AI: Artificial intelligence
API: Application programming interface
CDSS: Clinical Decision Support System
FlanT5: FlanT5-XXL model
GPT: Generative pre-trained transformer
Mixtral: Mixtral-8 × 7B-Instruct v0.1 model
ML: Machine learning
LLM: Large language model
OHNC: OpenHermes-2.5-neural-chat-7b-v3-1-7B model
Platypus 2: Platypus2-70B-Instruct model
ROC: Receiver operating characteristic
SLR: Systematic literature review
