# Supplementary material for: Title and abstract screening for literature reviews using large language models: an exploratory study in the biomedical domain
Source: Syst Rev. 2024 Jun 15;13:158. doi: 10.1186/s13643-024-02575-4 (PMC11180407; doi:10.1186/s13643-024-02575-4)
Supplement: Supplementary file 1 — Supplementary Material 1: Appendix 1: Sample prompt. [file 13643_2024_2575_MOESM1_ESM.docx]

An example for a prompt is shown in the following for the publication of Seessle et al. (1) which was included in the final analysis of the SLR of Appenzeller-Herzog et al. (2).

*“*

**[Instruction]**

*On a scale from 1 (very low probability) to 5 (very high probability), how would you consider the relevance of the following scientific publication to be included in a systematic literature review based on the inclusion criteria and based on title and abstract?*

**[Title of publication]**

*Title: Concomitant immune-related events in Wilson disease: implications for monitoring chelator therapy*

**[Abstract of publication]**

*, Abstract: BACKGROUND AND AIMS: Current guidelines favor the use of chelating agents (d-penicillamine, trientine) in first line therapy of symptomatic Wilson disease patients. Development of chelator induced immunological adverse events are a concern especially under d-penicillamine therapy. This study assessed the prevalence of co-existing or therapy-related immune-mediated diseases in Wilson disease patients, and evaluated the role of antinuclear antibodies in therapy monitoring. METHODS: We retrospectively analyzed 235 Wilson disease patients. Medical regimens were classified and analyzed in relation to adverse events and antinuclear antibody courses. RESULTS: Coexisting immune-mediated diseases were evident in 19/235 (8.1%) patients, of which 13/235 (5.5%) had pre-existing autoimmune diseases. Six patients (2.6%) developed an autoimmune disease under therapy, all of them under long-term d-penicillamine treatment. Data relating to antinuclear antibody courses during treatment and adverse events were available for patients treated with d-penicillamine (n=91), trientine (n=58), and zinc salts (n=58). No significant increase in antinuclear antibody titers in patients treated with d-penicillamine (16/91; 17.6%), trientine (12/58; 20.7%), and zinc (7/58; 12.1%) were found. CONCLUSION: Under long-term d-penicillamine therapy a minority of patients developed immune-mediated disease. Elevations in antinuclear antibodies were found frequently, but no correlations were evident between increases in antinuclear antibodies and the development of immune-mediated diseases or medical regimes. Thus, the value of antinuclear antibodies for monitoring adverse events under chelator therapy seems to be limited.*

**[Relevant Criteria]**

*, Relevant criteria:*

*-Patients with Wilson's Disease of any age or stage*

*-Study drug has to be one of four established therapies, namely DPen, trientine, TTM or Zn.*

*-Control could be placebo, no treatment or any other treatment that does not include the respective study drug*

*-Concomitant therapies had to be identical in the compared treatment arms*

*-Combination therapy regimens that include the respective monotherapy drug are not considered*

*-Prospective or retrospective studies reported*

*-Randomized, non-randomized controlled trials and comparative observational studies*

*-Animal studies, case reports, case series, cross‐sectional studies, before‐after studies, reviews, letters, abstract‐only publications, editorials, diagnostic or other testing studies and non‐controlled studies are excluded*

*”*

1. Seessle J, Gotthardt DN, Schäfer M, Gohdes A, Pfeiffenberger J, Ferenci P, et al. Concomitant immune-related events in Wilson disease: implications for monitoring chelator therapy. J Inherit Metab Dis. 2016 Jan;39(1):125–30.

2. Appenzeller‐Herzog C, Mathes T, Heeres MLS, Weiss KH, Houwen RHJ, Ewald H. Comparative effectiveness of common therapies for Wilson disease: A systematic review and meta‐analysis of controlled studies. Liver Int. 2019 Nov;39(11):2136–52.
