# Supplementary material for: Title and abstract screening for literature reviews using large language models: an exploratory study in the biomedical domain
Source: Syst Rev. 2024 Jun 15;13:158. doi: 10.1186/s13643-024-02575-4 (PMC11180407; doi:10.1186/s13643-024-02575-4)
Supplement: Supplementary file 2 — Supplementary Material 2: Appendix 2: Relevant criteria of published datasets. [file 13643_2024_2575_MOESM2_ESM.docx]

**Relevant Criteria strings**

The following contains the [Relevant criteria] strings for the published dataset. They were created upon agreement of the researchers FD, NC and PMP based on the descriptions in the publications of the corresponding SLRs.

**Appenzeller-Herzog_2020**

*“””*

*-Patients with Wilson's Disease of any age or stage*

*-Study drug has to be one of four established therapies, namely DPen, trientine, TTM or Zn.*

*-Control could be placebo, no treatment or any other treatment that does not include the respective study drug*

*-Concomitant therapies had to be identical in the compared treatment arms*

*-Combination therapy regimens that include the respective monotherapy drug are not considered*

*-Prospective or retrospective studies reported*

*-Randomized, non-randomized controlled trials and comparative observational studies*

*-Animal studies, case reports, case series, cross‐sectional studies, before‐after studies, reviews, letters, abstract‐only publications, editorials,*

*diagnostic or other testing studies and non‐controlled studies are excluded*

*”””*

based on Appenzeller-Herzog et al. (1).

**Bos_2018**

*"""*

*Inclusion criteria: (1) all studies have to be prospective*

*population-based cohort studies. Inherent to the nature of*

*population-based studies this means that except for a*

*selection criterion of age, no other selection criteria are*

*applied; (2) cerebral imaging had to be performed*

*(either magnetic resonance imaging [MRI] or computed*

*tomography) for the visualization of white matter*

*hyperintensities, covert brain infarcts, or microbleeds; (3) all*

*studies have to have investigated the association of any of the*

*three pathologies with the risk of incident all-dementia or*

*Alzheimer’s disease.*

*"""*

based on Bose et al. (2).

**Donners_2021**

*"""*

*The following inclusion criteria are applied: emicizumab*

*studies providing (1) data on humans, (2) original PK data or*

*modeled PK data or PK/PD relationships, and (3) access to*

*the abstract and the full text in English. In the event of doubt*

*regarding eligibility, the records or articles should be included.*

*"""*

based on Donners et al. (3).

**Jeyaraman_2021**

*"""*

*Inclusion Criteria*

*Studies are included if they meet the following PICOS criteria:*

*Population: Patients with knee osteoarthritis*

*Intervention: MSC therapy*

*Comparator: Usual care*

*Outcomes: Visual Analog Score (VAS) for Pain, Western Ontario McMaster Universities Osteoarthritis Index (WOMAC), Lysholm Knee Scale (Lysholm), Whole-Organ Magnetic Resonance Imaging Score (WORMS), Knee Osteoarthritis Outcome Score (KOOS), and adverse events*

*Study Design: Randomized controlled trials*

*Exclusion Criteria*

*Trials are excluded if they had the following characteristics:*

*1. Observational studies and interventional studies without a comparator group*

*2. Animal studies involving stem cell therapy for knee osteoarthritis models*

*3. Review*

*"""*

based on Jeyaraman et al. (4).

**Leenaars_2020**

*"""*

*Inclusion of all studies in which methotrexat (MTX) was administered to rheumatoid arthritis (RA) patients or RA animal models.*

*Exclusion of studies on other diseases than RA, studies that were not in vivo,*

*studies that did not administer MTX (or only had treatment groups co-administering MTX with other*

*experimental drugs), studies that did not analyse efficacy (e.g., safety studies), and publications that*

*do not contain new data (e.g., reviews and editorials).*

*"""*

based on Leenaars et al. (5).

**Meijboom_2021**

*"""*

*Articles are included if they meet the*

*following criteria: (1) study involved transitioning from a*

*TNF-alpha inhibitor (including etanercept, infliximab, and adalimumab)*

*originator to a biosimilar, (2) the number of patients*

*who retransitioned is reported or can be calculated, (3)*

*the article is an original research article published in a*

*peer-reviewed journal, (4) the article included baseline*

*characteristics of the patients who transitioned, (5) the article*

*is written in English. Transitioning is defined as*

*patients in whom the biosimilar was introduced after the*

*originator, without treatment with other drugs in between.*

*Retransitioning is defined as restarting the originator*

*directly after discontinuing a biosimilar, without treatment*

*with other drugs in between. In summary, transitioning is*

*defined as switching from the originator to a biosimilar;*

*retransitioning is defined as switching from the originator*

*to a biosimilar and back to the originator. Both transitioning*

*and retransitioning involve changes with the same active*

*biological substance.*

*"""*

based on Meijboom et al. (6).

**Muthu_2021**

*"""*

*Inclusion Criteria*

*To be included, a study should meet the*

*following criteria:*

*1. The study should be an RCT with 1:1 parallel twoarm*

*design.*

*2. The study must be related to spine surgery involving*

*preoperative or intraoperative or postoperative variables.*

*3. The study must have a dichotomous primary or*

*secondary outcome.*

*Exclusion Criteria*

*1. Studies not involving human subjects.*

*2. Studies with continuous variable outcomes like pain*

*scores, Oswestry Disability Index scores, time to*

*union without predefined clinical success criteria.*

*3. Studies that did not report a statistically significant*

*primary or secondary outcome measure.*

*"""*

based on Muthu et al. (7).

**Oud_2018**

*"""*

*Randomized controlled trials (RCT)s on four specialized psychotherapies*

*(DBT: dialectic behavior therapy, MBT: mentalization-based treatment, TFP: transference-focused therapy and ST: schema therapy)*

*for adults (18 years and older)*

*with Borderline personality disorder (BPD), which includes an individual psychotherapy*

*component and had a duration of 16 weeks or more.*

*Eligible comparison groups are other protocolized and*

*specialized psychotherapies, or control groups, for*

*example, treatment as usual (TAU), waiting list, attention*

*control or community treatment by experts (CTBE).*

*Studies are excluded with a cut-off of <66% of the participants*

*having BPD, unless disaggregated data are provided. Studies are excluded that tested incomplete*

*versions of specialized treatment, for example,*

*studies that investigated only skills training instead of*

*the full DBT program.*

*"""*

based on Oud et al. (8).

**Van_de_Schoot_2018**

*"""*

*(a) longitudinal studies with at least three measurement*

*waves measuring posttraumatic stress disorder (PTSD), (b) studies that measured PTSD*

*on a continuous scale via an interviewor questionnaire, (c) and studies*

*that used a clustering method (Latent growth mixture modelling, hierarchical*

*cluster analysis), (d) traumatic stress symptoms following events*

*that appeared to fulfill DSM-IV criterion A1 for PTSD or acute*

*stress disorder.*

*"""*

based on van de Schoot et al. (9)

**Wolters_2019**

*"""*

*-Prospective studies of humans that report the risk of all-cause dementia or Alzheimer's disease in relation to coronary heart disease or congestive heart failure*

*-Only original articles*

*"""*

based on Wolters et al. (10).

1. Appenzeller‐Herzog C, Mathes T, Heeres MLS, Weiss KH, Houwen RHJ, Ewald H. Comparative effectiveness of common therapies for Wilson disease: A systematic review and meta‐analysis of controlled studies. Liver Int. 2019 Nov;39(11):2136–52.

2. Bos D, Wolters FJ, Darweesh SKL, Vernooij MW, De Wolf F, Ikram MA, et al. Cerebral small vessel disease and the risk of dementia: A systematic review and meta‐analysis of population‐based evidence. Alzheimer’s &amp; Dementia. 2018 Nov;14(11):1482–92.

3. Donners AAMT, Rademaker CMA, Bevers LAH, Huitema ADR, Schutgens REG, Egberts TCG, et al. Pharmacokinetics and Associated Efficacy of Emicizumab in Humans: A Systematic Review. Clin Pharmacokinet. 2021 Nov;60(11):1395–406.

4. Jeyaraman M, Muthu S, Ganie PA. Does the Source of Mesenchymal Stem Cell Have an Effect in the Management of Osteoarthritis of the Knee? Meta-Analysis of Randomized Controlled Trials. CARTILAGE. 2021 Dec;13(1_suppl):1532S-1547S.

5. Leenaars C, Stafleu F, De Jong D, Van Berlo M, Geurts T, Coenen-de Roo T, et al. A Systematic Review Comparing Experimental Design of Animal and Human Methotrexate Efficacy Studies for Rheumatoid Arthritis: Lessons for the Translational Value of Animal Studies. Animals. 2020 Jun 17;10(6):1047.

6. Meijboom RW, Gardarsdottir H, Egberts TCG, Giezen TJ. Patients Retransitioning from Biosimilar TNFα Inhibitor to the Corresponding Originator After Initial Transitioning to the Biosimilar: A Systematic Review. BioDrugs. 2022 Jan;36(1):27–39.

7. Muthu S, Ramakrishnan E. Fragility Analysis of Statistically Significant Outcomes of Randomized Control Trials in Spine Surgery: A Systematic Review. Spine. 2021 Feb 1;46(3):198–208.

8. Oud M, Arntz A, Hermens ML, Verhoef R, Kendall T. Specialized psychotherapies for adults with borderline personality disorder: A systematic review and meta-analysis. Aust N Z J Psychiatry. 2018 Oct;52(10):949–61.

9. Van De Schoot R, Sijbrandij M, Depaoli S, Winter SD, Olff M, Van Loey NE. Bayesian PTSD-Trajectory Analysis with Informed Priors Based on a Systematic Literature Search and Expert Elicitation. Multivariate Behavioral Research. 2018 Mar 4;53(2):267–91.

10. Wolters FJ, Segufa RA, Darweesh SKL, Bos D, Ikram MA, Sabayan B, et al. Coronary heart disease, heart failure, and the risk of dementia: A systematic review and meta‐analysis. Alzheimer’s &amp; Dementia. 2018 Nov;14(11):1493–504.
