# Supplementary material for: Title and abstract screening for literature reviews using large language models: an exploratory study in the biomedical domain
Source: Syst Rev. 2024 Jun 15;13:158. doi: 10.1186/s13643-024-02575-4 (PMC11180407; doi:10.1186/s13643-024-02575-4)
Supplement: Supplementary file 3 — Supplementary Material 3: Appendix 3: Performance of models on data sets. [file 13643_2024_2575_MOESM3_ESM.docx]

**Performance of models on data sets**

| **Appenzeller-Herzog_2020** |  | Total publications | 3479 |  |
| --- | --- | --- | --- | --- |
| Relevant publications | 26 | Irrelevant publications | 3453 |  |
|  |  |  |  |  |
| **FlanT5** |  |  |  |  |
| Publications with invalid classification | 0 |  |  |  |
| **Threshold for classifier** | **5** | **4+** | **3+** | **2+** |
| **True positive** | 0 | 24 | 24 | 26 |
| **True negative** | 3453 | 2078 | 2078 | 27 |
| **False positive** | 0 | 1375 | 1375 | 3426 |
| **False negative** | 26 | 2 | 2 | 0 |
| **Sensitivity (=Recall)** | 0.00% | 46.15% | 92.31% | 100.00% |
| **Specificity** | 100.00% | 60.18% | 60.18% | 0.78% |
| **Accuracy** | 99.25% | 60.42% | 60.42% | 1.52% |
| **Precision** | - | 1.72% | 1.72% | 0.75% |
| **F1-Score** | - | 3.37% | 3.37% | 1.50% |
|  |  |  |  |  |
| **OHNC** |  |  |  |  |
| Publications with invalid classification | 0 |  |  |  |
| **Threshold for classifier** | **5** | **4+** | **3+** | **2+** |
| **True positive** | 0 | 19 | 25 | 26 |
| **True negative** | 3453 | 2942 | 973 | 118 |
| **False positive** | 0 | 511 | 2480 | 3335 |
| **False negative** | 26 | 7 | 1 | 0 |
| **Sensitivity (=Recall)** | 0.00% | 73.08% | 96.15% | 100.00% |
| **Specificity** | 100.00% | 85.20% | 28.18% | 3.42% |
| **Accuracy** | 99.25% | 85.11% | 28.69% | 4.14% |
| **Precision** | - | 3.58% | 1.00% | 0.77% |
| **F1-Score** | - | 6.83% | 1.98% | 1.54% |
|  |  |  |  |  |
| **Mixtral** |  |  |  |  |
| Publications with invalid classification | 0 |  |  |  |
| **Threshold for classifier** | **5** | **4+** | **3+** | **2+** |
| **True positive** | 9 | 18 | 21 | 24 |
| **True negative** | 3088 | 2735 | 2478 | 1717 |
| **False positive** | 365 | 718 | 975 | 1736 |
| **False negative** | 17 | 8 | 5 | 2 |
| **Sensitivity (=Recall)** | 34.62% | 69.23% | 80.77% | 92.31% |
| **Specificity** | 89.43% | 79.21% | 71.76% | 49.72% |
| **Accuracy** | 89.02% | 79.13% | 71.83% | 50.04% |
| **Precision** | 2.41% | 2.45% | 2.11% | 1.36% |
| **F1-Score** | 4.50% | 4.72% | 4.11% | 2.69% |
|  |  |  |  |  |
| **Platypus 2** |  |  |  |  |
| Publications with invalid classification | 0 |  |  |  |
| **Threshold for classifier** | **5** | **4+** | **3+** | **2+** |
| **True positive** | 0 | 12 | 24 | 26 |
| **True negative** | 3453 | 3287 | 1681 | 3 |
| **False positive** | 0 | 166 | 1772 | 3450 |
| **False negative** | 26 | 14 | 2 | 0 |
| **Sensitivity (=Recall)** | 0.00% | 46.15% | 92.31% | 100.00% |
| **Specificity** | 100.00% | 95.19% | 48.68% | 0.09% |
| **Accuracy** | 99.25% | 94.83% | 48.83% | 0.14% |
| **Precision** | - | 6.74% | 0.67% | 0.06% |
| **F1-Score** | - | 11.76% | 1.34% | 0.12% |
|  |  |  |  |  |
| **Bos_2018** |  | Total publications | 5756 |  |
| Relevant publications | 10 | Irrelevant publications | 5746 |  |
|  |  |  |  |  |
| **FlanT5** |  |  |  |  |
| Publications with invalid classification | 0 |  |  |  |
| **Threshold for classifier** | **5** | **4+** | **3+** | **2+** |
| **True positive** | 0 | 10 | 10 | 10 |
| **True negative** | 5746 | 1400 | 1399 | 0 |
| **False positive** | 0 | 4346 | 4347 | 5746 |
| **False negative** | 10 | 0 | 0 | 0 |
| **Sensitivity (=Recall)** | 0.00% | 100.00% | 100.00% | 100.00% |
| **Specificity** | 100.00% | 24.36% | 24.35% | 0.00% |
| **Accuracy** | 99.83% | 24.50% | 24.48% | 0.17% |
| **Precision** | - | 0.23% | 0.23% | 0.17% |
| **F1-Score** | - | 0.46% | 0.46% | 0.35% |
|  |  |  |  |  |
| **OHNC** |  |  |  |  |
| Publications with invalid classification | 0 |  |  |  |
| **Threshold for classifier** | **5** | **4+** | **3+** | **2+** |
| **True positive** | 0 | 9 | 10 | 10 |
| **True negative** | 5746 | 4920 | 1382 | 135 |
| **False positive** | 0 | 826 | 4364 | 5611 |
| **False negative** | 10 | 1 | 0 | 0 |
| **Sensitivity (=Recall)** | 0.00% | 90.00% | 100.00% | 100.00% |
| **Specificity** | 100.00% | 85.62% | 24.05% | 2.35% |
| **Accuracy** | 99.83% | 85.63% | 24.18% | 2.52% |
| **Precision** | - | 1.08% | 0.23% | 0.18% |
| **F1-Score** | - | 2.13% | 0.46% | 0.36% |
|  |  |  |  |  |
| **Mixtral** |  |  |  |  |
| Publications with invalid classification | 0 |  |  |  |
| **Threshold for classifier** | **5** | **4+** | **3+** | **2+** |
| **True positive** | 7 | 9 | 9 | 10 |
| **True negative** | 5707 | 5620 | 5561 | 4322 |
| **False positive** | 39 | 126 | 185 | 1424 |
| **False negative** | 3 | 1 | 1 | 0 |
| **Sensitivity (=Recall)** | 70.00% | 90.00% | 90.00% | 100.00% |
| **Specificity** | 99.32% | 97.81% | 96.78% | 75.22% |
| **Accuracy** | 99.27% | 97.79% | 96.77% | 75.26% |
| **Precision** | 15.22% | 6.67% | 4.64% | 0.70% |
| **F1-Score** | 25.00% | 12.41% | 8.82% | 1.39% |
|  |  |  |  |  |
| **Platypus 2** |  |  |  |  |
| Publications with invalid classification | 0 |  |  |  |
| **Threshold for classifier** | **5** | **4+** | **3+** | **2+** |
| **True positive** | 0 | 9 | 10 | 10 |
| **True negative** | 5746 | 5432 | 2786 | 19 |
| **False positive** | 0 | 314 | 2960 | 5727 |
| **False negative** | 10 | 1 | 0 | 0 |
| **Sensitivity (=Recall)** | 0.00% | 90.00% | 100.00% | 100.00% |
| **Specificity** | 100.00% | 94.54% | 48.49% | 0.33% |
| **Accuracy** | 99.83% | 94.53% | 48.58% | 0.50% |
| **Precision** | - | 2.79% | 0.34% | 0.17% |
| **F1-Score** | - | 5.41% | 0.67% | 0.35% |
|  |  |  |  |  |
| **Donners_2020** |  | Total publications | 660 |  |
| Relevant publications | 15 | Irrelevant publications | 645 |  |
|  |  |  |  |  |
| **FlanT5** |  |  |  |  |
| Publications with invalid classification | 0 |  |  |  |
| **Threshold for classifier** | **5** | **4+** | **3+** | **2+** |
| **True positive** | 0 | 14 | 14 | 15 |
| **True negative** | 645 | 107 | 107 | 1 |
| **False positive** | 0 | 538 | 538 | 644 |
| **False negative** | 15 | 1 | 1 | 0 |
| **Sensitivity (=Recall)** | 0.00% | 93.33% | 93.33% | 100.00% |
| **Specificity** | 100.00% | 16.59% | 16.59% | 0.16% |
| **Accuracy** | 97.73% | 18.33% | 18.33% | 2.42% |
| **Precision** | - | 2.54% | 2.54% | 2.28% |
| **F1-Score** | - | 4.94% | 4.94% | 4.45% |
|  |  |  |  |  |
| **OHNC** |  |  |  |  |
| Publications with invalid classification | 0 |  |  |  |
| **Threshold for classifier** | **5** | **4+** | **3+** | **2+** |
| **True positive** | 0 | 12 | 15 | 15 |
| **True negative** | 640 | 227 | 18 | 4 |
| **False positive** | 5 | 418 | 627 | 641 |
| **False negative** | 15 | 3 | 0 | 0 |
| **Sensitivity (=Recall)** | 0.00% | 80.00% | 100.00% | 100.00% |
| **Specificity** | 99.22% | 35.19% | 2.79% | 0.62% |
| **Accuracy** | 96.97% | 36.21% | 5.00% | 2.88% |
| **Precision** | 0.00% | 2.79% | 2.34% | 2.29% |
| **F1-Score** | - | 5.39% | 4.57% | 4.47% |
|  |  |  |  |  |
| **Mixtral** |  |  |  |  |
| Publications with invalid classification | 4 |  |  |  |
| **Threshold for classifier** | **5** | **4+** | **3+** | **2+** |
| **True positive** | 13 | 13 | 14 | 15 |
| **True negative** | 385 | 228 | 174 | 87 |
| **False positive** | 260 | 417 | 471 | 558 |
| **False negative** | 2 | 2 | 1 | 0 |
| **Sensitivity (=Recall)** | 86.67% | 86.67% | 93.33% | 100.00% |
| **Specificity** | 59.69% | 35.35% | 26.98% | 13.49% |
| **Accuracy** | 60.30% | 36.52% | 28.48% | 15.45% |
| **Precision** | 4.76% | 3.02% | 2.89% | 2.62% |
| **F1-Score** | 9.03% | 5.84% | 5.60% | 5.10% |
|  |  |  |  |  |
| **Platypus 2** |  |  |  |  |
| Publications with invalid classification | 0 |  |  |  |
| **Threshold for classifier** | **5** | **4+** | **3+** | **2+** |
| **True positive** | 0 | 15 | 15 | 15 |
| **True negative** | 645 | 128 | 63 | 0 |
| **False positive** | 0 | 517 | 582 | 645 |
| **False negative** | 15 | 0 | 0 | 0 |
| **Sensitivity (=Recall)** | 0.00% | 100.00% | 100.00% | 100.00% |
| **Specificity** | 100.00% | 19.84% | 9.77% | 0.00% |
| **Accuracy** | 97.73% | 21.67% | 11.82% | 2.27% |
| **Precision** | - | 2.82% | 2.51% | 2.27% |
| **F1-Score** | - | 5.48% | 4.90% | 4.44% |
|  |  |  |  |  |
| **Jeyaraman_2021** |  | Total publications | 1194 |  |
| Relevant publications | 96 | Irrelevant publications | 5746 |  |
|  |  |  |  |  |
| **FlanT5** |  |  |  |  |
| Publications with invalid classification | 0 |  |  |  |
| **Threshold for classifier** | **5** | **4+** | **3+** | **2+** |
| **True positive** | 0 | 85 | 85 | 96 |
| **True negative** | 1098 | 293 | 290 | 0 |
| **False positive** | 0 | 805 | 808 | 1098 |
| **False negative** | 96 | 11 | 11 | 0 |
| **Sensitivity (=Recall)** | 0.00% | 88.54% | 88.54% | 100.00% |
| **Specificity** | 100.00% | 26.68% | 26.41% | 0.00% |
| **Accuracy** | 91.96% | 31.66% | 31.41% | 8.04% |
| **Precision** | - | 9.55% | 9.52% | 8.04% |
| **F1-Score** | - | 17.24% | 17.19% | 14.88% |
|  |  |  |  |  |
| **OHNC** |  |  |  |  |
| Publications with invalid classification | 0 |  |  |  |
| **Threshold for classifier** | **5** | **4+** | **3+** | **2+** |
| **True positive** | 0 | 90 | 96 | 96 |
| **True negative** | 1097 | 275 | 17 | 1 |
| **False positive** | 1 | 823 | 1081 | 1097 |
| **False negative** | 96 | 6 | 0 | 0 |
| **Sensitivity (=Recall)** | 0.00% | 93.75% | 100.00% | 100.00% |
| **Specificity** | 99.91% | 25.05% | 1.55% | 0.09% |
| **Accuracy** | 91.88% | 30.57% | 9.46% | 8.12% |
| **Precision** | 0.00% | 9.86% | 8.16% | 8.05% |
| **F1-Score** | - | 17.84% | 15.08% | 14.90% |
|  |  |  |  |  |
| **Mixtral** |  |  |  |  |
| Publications with invalid classification | 0 |  |  |  |
| **Threshold for classifier** | **5** | **4+** | **3+** | **2+** |
| **True positive** | 4 | 14 | 23 | 73 |
| **True negative** | 1090 | 1067 | 1039 | 626 |
| **False positive** | 8 | 31 | 59 | 472 |
| **False negative** | 92 | 82 | 73 | 23 |
| **Sensitivity (=Recall)** | 4.17% | 14.58% | 23.96% | 76.04% |
| **Specificity** | 99.27% | 97.18% | 94.63% | 57.01% |
| **Accuracy** | 91.62% | 90.54% | 88.94% | 58.54% |
| **Precision** | 33.33% | 31.11% | 28.05% | 13.39% |
| **F1-Score** | 7.41% | 19.86% | 25.84% | 22.78% |
|  |  |  |  |  |
| **Platypus 2** |  |  |  |  |
| Publications with invalid classification | 0 |  |  |  |
| **Threshold for classifier** | **5** | **4+** | **3+** | **2+** |
| **True positive** | 0 | 50 | 96 | 96 |
| **True negative** | 1098 | 971 | 179 | 0 |
| **False positive** | 0 | 127 | 919 | 1098 |
| **False negative** | 96 | 46 | 0 | 0 |
| **Sensitivity (=Recall)** | 0.00% | 52.08% | 100.00% | 100.00% |
| **Specificity** | 100.00% | 88.43% | 16.30% | 0.00% |
| **Accuracy** | 91.96% | 85.51% | 23.03% | 8.04% |
| **Precision** | - | 28.25% | 9.46% | 8.04% |
| **F1-Score** | - | 36.63% | 17.28% | 14.88% |
|  |  |  |  |  |
| **Leenaars_2020** |  | Total publications | 9543 |  |
| Relevant publications | 792 | Irrelevant publications | 8751 |  |
|  |  |  |  |  |
| **FlanT5** |  |  |  |  |
| Publications with invalid classification | 0 |  |  |  |
| **Threshold for classifier** | **5** | **4+** | **3+** | **2+** |
| **True positive** | 0 | 783 | 783 | 792 |
| **True negative** | 8751 | 2942 | 2942 | 3 |
| **False positive** | 0 | 5809 | 5809 | 8748 |
| **False negative** | 792 | 9 | 9 | 0 |
| **Sensitivity (=Recall)** | 0.00% | 98.86% | 98.86% | 100.00% |
| **Specificity** | 100.00% | 33.62% | 33.62% | 0.03% |
| **Accuracy** | 91.70% | 39.03% | 39.03% | 8.33% |
| **Precision** | - | 11.88% | 11.88% | 8.30% |
| **F1-Score** | - | 21.21% | 21.21% | 15.33% |
|  |  |  |  |  |
| **OHNC** |  |  |  |  |
| Publications with invalid classification | 0 |  |  |  |
| **Threshold for classifier** | **5** | **4+** | **3+** | **2+** |
| **True positive** | 0 | 752 | 791 | 792 |
| **True negative** | 8737 | 4697 | 1158 | 187 |
| **False positive** | 14 | 4054 | 7593 | 8564 |
| **False negative** | 792 | 40 | 1 | 0 |
| **Sensitivity (=Recall)** | 0.00% | 94.95% | 99.87% | 100.00% |
| **Specificity** | 99.84% | 53.67% | 13.23% | 2.14% |
| **Accuracy** | 91.55% | 57.10% | 20.42% | 10.26% |
| **Precision** | 0.00% | 15.65% | 9.43% | 8.47% |
| **F1-Score** | - | 26.87% | 17.24% | 15.61% |
|  |  |  |  |  |
| **Mixtral** |  |  |  |  |
| Publications with invalid classification | 13 |  |  |  |
| **Threshold for classifier** | **5** | **4+** | **3+** | **2+** |
| **True positive** | 562 | 686 | 712 | 784 |
| **True negative** | 6896 | 5977 | 5685 | 3569 |
| **False positive** | 1855 | 2774 | 3066 | 5182 |
| **False negative** | 230 | 106 | 80 | 8 |
| **Sensitivity (=Recall)** | 70.96% | 86.62% | 89.90% | 98.99% |
| **Specificity** | 78.80% | 68.30% | 64.96% | 40.78% |
| **Accuracy** | 78.15% | 69.82% | 67.03% | 45.61% |
| **Precision** | 23.25% | 19.83% | 18.85% | 13.14% |
| **F1-Score** | 35.03% | 32.27% | 31.16% | 23.20% |
|  |  |  |  |  |
| **Platypus 2** |  |  |  |  |
| Publications with invalid classification | 0 |  |  |  |
| **Threshold for classifier** | **5** | **4+** | **3+** | **2+** |
| **True positive** | 0 | 732 | 791 | 792 |
| **True negative** | 8751 | 5795 | 2627 | 3 |
| **False positive** | 0 | 2956 | 6124 | 8748 |
| **False negative** | 792 | 60 | 1 | 0 |
| **Sensitivity (=Recall)** | 0.00% | 92.42% | 99.87% | 100.00% |
| **Specificity** | 100.00% | 66.22% | 30.02% | 0.03% |
| **Accuracy** | 91.70% | 68.40% | 35.82% | 8.33% |
| **Precision** | - | 19.85% | 11.44% | 8.30% |
| **F1-Score** | - | 32.68% | 20.53% | 15.33% |
|  |  |  |  |  |
| **Mejboom_2021** |  | Total publications | 2224 |  |
| Relevant publications | 37 | Irrelevant publications | 2187 |  |
|  |  |  |  |  |
| **FlanT5** |  |  |  |  |
| Publications with invalid classification | 0 |  |  |  |
| **Threshold for classifier** | **5** | **4+** | **3+** | **2+** |
| **True positive** | 0 | 37 | 37 | 37 |
| **True negative** | 2187 | 348 | 348 | 0 |
| **False positive** | 0 | 1839 | 1839 | 2187 |
| **False negative** | 37 | 0 | 0 | 0 |
| **Sensitivity (=Recall)** | 0.00% | 100.00% | 100.00% | 100.00% |
| **Specificity** | 100.00% | 15.91% | 15.91% | 0.00% |
| **Accuracy** | 98.34% | 17.31% | 17.31% | 1.66% |
| **Precision** | - | 1.97% | 1.97% | 1.66% |
| **F1-Score** | - | 3.87% | 3.87% | 3.27% |
|  |  |  |  |  |
| **OHNC** |  |  |  |  |
| Publications with invalid classification | 0 |  |  |  |
| **Threshold for classifier** | **5** | **4+** | **3+** | **2+** |
| **True positive** | 0 | 35 | 37 | 37 |
| **True negative** | 2187 | 959 | 90 | 1 |
| **False positive** | 0 | 1228 | 2097 | 2186 |
| **False negative** | 37 | 2 | 0 | 0 |
| **Sensitivity (=Recall)** | 0.00% | 94.59% | 100.00% | 100.00% |
| **Specificity** | 100.00% | 43.85% | 4.12% | 0.05% |
| **Accuracy** | 98.34% | 44.69% | 5.71% | 1.71% |
| **Precision** | - | 2.77% | 1.73% | 1.66% |
| **F1-Score** | - | 5.38% | 3.41% | 3.27% |
|  |  |  |  |  |
| **Mixtral** |  |  |  |  |
| Publications with invalid classification | 0 |  |  |  |
| **Threshold for classifier** | **5** | **4+** | **3+** | **2+** |
| **True positive** | 28 | 36 | 36 | 36 |
| **True negative** | 1857 | 1524 | 1324 | 650 |
| **False positive** | 330 | 663 | 863 | 1537 |
| **False negative** | 9 | 1 | 1 | 1 |
| **Sensitivity (=Recall)** | 75.68% | 97.30% | 97.30% | 97.30% |
| **Specificity** | 84.91% | 69.68% | 60.54% | 29.72% |
| **Accuracy** | 84.76% | 70.14% | 61.15% | 30.85% |
| **Precision** | 7.82% | 5.15% | 4.00% | 2.29% |
| **F1-Score** | 14.18% | 9.78% | 7.69% | 4.47% |
|  |  |  |  |  |
| **Platypus 2** |  |  |  |  |
| Publications with invalid classification | 0 |  |  |  |
| **Threshold for classifier** | **5** | **4+** | **3+** | **2+** |
| **True positive** | 0 | 32 | 37 | 37 |
| **True negative** | 2187 | 1522 | 842 | 5 |
| **False positive** | 0 | 665 | 1345 | 2182 |
| **False negative** | 37 | 5 | 0 | 0 |
| **Sensitivity (=Recall)** | 0.00% | 86.49% | 100.00% | 100.00% |
| **Specificity** | 100.00% | 69.59% | 38.50% | 0.23% |
| **Accuracy** | 98.34% | 69.87% | 39.52% | 1.89% |
| **Precision** | - | 4.59% | 2.68% | 1.67% |
| **F1-Score** | - | 8.72% | 5.21% | 3.28% |
|  |  |  |  |  |
| **Muthu_2021** |  | Total publications | 3254 |  |
| Relevant publications | 354 | Irrelevant publications | 2900 |  |
|  |  |  |  |  |
| **FlanT5** |  |  |  |  |
| Publications with invalid classification | 0 |  |  |  |
| **Threshold for classifier** | **5** | **4+** | **3+** | **2+** |
| **True positive** | 0 | 336 | 337 | 354 |
| **True negative** | 2900 | 166 | 148 | 0 |
| **False positive** | 0 | 2734 | 2752 | 2900 |
| **False negative** | 354 | 18 | 17 | 0 |
| **Sensitivity (=Recall)** | 0.00% | 94.92% | 95.20% | 100.00% |
| **Specificity** | 100.00% | 5.72% | 5.10% | 0.00% |
| **Accuracy** | 89.12% | 15.43% | 14.90% | 10.88% |
| **Precision** | - | 10.94% | 10.91% | 10.88% |
| **F1-Score** | - | 19.63% | 19.58% | 19.62% |
|  |  |  |  |  |
| **OHNC** |  |  |  |  |
| Publications with invalid classification | 0 |  |  |  |
| **Threshold for classifier** | **5** | **4+** | **3+** | **2+** |
| **True positive** | 6 | 350 | 354 | 354 |
| **True negative** | 2890 | 636 | 55 | 1 |
| **False positive** | 10 | 2264 | 2845 | 2899 |
| **False negative** | 348 | 4 | 0 | 0 |
| **Sensitivity (=Recall)** | 1.69% | 98.87% | 100.00% | 100.00% |
| **Specificity** | 99.66% | 21.93% | 1.90% | 0.03% |
| **Accuracy** | 89.00% | 30.30% | 12.57% | 10.91% |
| **Precision** | 37.50% | 13.39% | 11.07% | 10.88% |
| **F1-Score** | 3.24% | 23.58% | 19.93% | 19.63% |
|  |  |  |  |  |
| **Mixtral** |  |  |  |  |
| Publications with invalid classification | 0 |  |  |  |
| **Threshold for classifier** | **5** | **4+** | **3+** | **2+** |
| **True positive** | 301 | 323 | 327 | 344 |
| **True negative** | 1791 | 1568 | 1456 | 709 |
| **False positive** | 1109 | 1332 | 1444 | 2191 |
| **False negative** | 53 | 31 | 27 | 10 |
| **Sensitivity (=Recall)** | 85.03% | 91.24% | 92.37% | 97.18% |
| **Specificity** | 61.76% | 54.07% | 50.21% | 24.45% |
| **Accuracy** | 64.29% | 58.11% | 54.79% | 32.36% |
| **Precision** | 21.35% | 19.52% | 18.46% | 13.57% |
| **F1-Score** | 34.13% | 32.16% | 30.78% | 23.81% |
|  |  |  |  |  |
| **Platypus 2** |  |  |  |  |
| Publications with invalid classification | 0 |  |  |  |
| **Threshold for classifier** | **5** | **4+** | **3+** | **2+** |
| **True positive** | 0 | 278 | 354 | 354 |
| **True negative** | 2900 | 1764 | 348 | 0 |
| **False positive** | 0 | 1136 | 2552 | 2900 |
| **False negative** | 354 | 76 | 0 | 0 |
| **Sensitivity (=Recall)** | 0.00% | 78.53% | 100.00% | 100.00% |
| **Specificity** | 100.00% | 60.83% | 12.00% | 0.00% |
| **Accuracy** | 89.12% | 62.75% | 21.57% | 10.88% |
| **Precision** | - | 19.66% | 12.18% | 10.88% |
| **F1-Score** | - | 31.45% | 21.72% | 19.62% |
|  |  |  |  |  |
| **Oud_2018** |  | Total publications | 20 |  |
| Relevant publications | 1053 | Irrelevant publications | 1033 |  |
|  |  |  |  |  |
| **FlanT5** |  |  |  |  |
| Publications with invalid classification | 0 |  |  |  |
| **Threshold for classifier** | **5** | **4+** | **3+** | **2+** |
| **True positive** | 0 | 20 | 20 | 20 |
| **True negative** | 1033 | 470 | 469 | 0 |
| **False positive** | 0 | 563 | 564 | 1033 |
| **False negative** | 20 | 0 | 0 | 0 |
| **Sensitivity (=Recall)** | 0.00% | 100.00% | 100.00% | 100.00% |
| **Specificity** | 100.00% | 45.50% | 45.40% | 0.00% |
| **Accuracy** | 98.10% | 46.53% | 46.44% | 1.90% |
| **Precision** | - | 3.43% | 3.42% | 1.90% |
| **F1-Score** | - | 6.63% | 6.62% | 3.73% |
|  |  |  |  |  |
| **OHNC** |  |  |  |  |
| Publications with invalid classification | 0 |  |  |  |
| **Threshold for classifier** | **5** | **4+** | **3+** | **2+** |
| **True positive** | 0 | 20 | 20 | 20 |
| **True negative** | 1033 | 884 | 304 | 18 |
| **False positive** | 0 | 149 | 729 | 1015 |
| **False negative** | 20 | 0 | 0 | 0 |
| **Sensitivity (=Recall)** | 0.00% | 100.00% | 100.00% | 100.00% |
| **Specificity** | 100.00% | 85.58% | 29.43% | 1.74% |
| **Accuracy** | 98.10% | 85.85% | 30.77% | 3.61% |
| **Precision** | - | 11.83% | 2.67% | 1.93% |
| **F1-Score** | - | 21.16% | 5.20% | 3.79% |
|  |  |  |  |  |
| **Mixtral** |  |  |  |  |
| Publications with invalid classification | 1 |  |  |  |
| **Threshold for classifier** | **5** | **4+** | **3+** | **2+** |
| **True positive** | 20 | 20 | 20 | 20 |
| **True negative** | 935 | 868 | 796 | 565 |
| **False positive** | 98 | 165 | 237 | 468 |
| **False negative** | 0 | 0 | 0 | 0 |
| **Sensitivity (=Recall)** | 100.00% | 100.00% | 100.00% | 100.00% |
| **Specificity** | 90.51% | 84.03% | 77.06% | 54.70% |
| **Accuracy** | 90.69% | 84.33% | 77.49% | 55.56% |
| **Precision** | 16.95% | 10.81% | 7.78% | 4.10% |
| **F1-Score** | 28.99% | 19.51% | 14.44% | 7.87% |
|  |  |  |  |  |
| **Platypus 2** |  |  |  |  |
| Publications with invalid classification | 0 |  |  |  |
| **Threshold for classifier** | **5** | **4+** | **3+** | **2+** |
| **True positive** | 0 | 20 | 20 | 20 |
| **True negative** | 1033 | 912 | 412 | 1 |
| **False positive** | 0 | 121 | 621 | 1032 |
| **False negative** | 20 | 0 | 0 | 0 |
| **Sensitivity (=Recall)** | 0.00% | 100.00% | 100.00% | 100.00% |
| **Specificity** | 100.00% | 88.29% | 39.88% | 0.10% |
| **Accuracy** | 98.10% | 88.51% | 41.03% | 1.99% |
| **Precision** | - | 14.18% | 3.12% | 1.90% |
| **F1-Score** | - | 24.84% | 6.05% | 3.73% |
|  |  |  |  |  |
| **van_de_Schoot_**  **2018** |  | Total publications | 6225 |  |
| Relevant publications | 38 | Irrelevant publications | 6187 |  |
|  |  |  |  |  |
| **FlanT5** |  |  |  |  |
| Publications with invalid classification | 0 |  |  |  |
| **Threshold for classifier** | **5** | **4+** | **3+** | **2+** |
| **True positive** | 0 | 36 | 36 | 38 |
| **True negative** | 6187 | 1340 | 1340 | 0 |
| **False positive** | 0 | 4847 | 4847 | 6187 |
| **False negative** | 38 | 2 | 2 | 0 |
| **Sensitivity (=Recall)** | 0.00% | 94.74% | 94.74% | 100.00% |
| **Specificity** | 100.00% | 21.66% | 21.66% | 0.00% |
| **Accuracy** | 99.39% | 22.10% | 22.10% | 0.61% |
| **Precision** | - | 0.74% | 0.74% | 0.61% |
| **F1-Score** | - | 1.46% | 1.46% | 1.21% |
|  |  |  |  |  |
| **OHNC** |  |  |  |  |
| Publications with invalid classification | 0 |  |  |  |
| **Threshold for classifier** | **5** | **4+** | **3+** | **2+** |
| **True positive** | 0 | 25 | 35 | 38 |
| **True negative** | 6186 | 4796 | 1258 | 110 |
| **False positive** | 1 | 1391 | 4929 | 6077 |
| **False negative** | 38 | 13 | 3 | 0 |
| **Sensitivity (=Recall)** | 0.00% | 65.79% | 92.11% | 100.00% |
| **Specificity** | 99.98% | 77.52% | 20.33% | 1.78% |
| **Accuracy** | 99.37% | 77.45% | 20.77% | 2.38% |
| **Precision** | 0.00% | 1.77% | 0.71% | 0.62% |
| **F1-Score** | - | 3.44% | 1.40% | 1.24% |
|  |  |  |  |  |
| **Mixtral** |  |  |  |  |
| Publications with invalid classification | 0 |  |  |  |
| **Threshold for classifier** | **5** | **4+** | **3+** | **2+** |
| **True positive** | 25 | 34 | 36 | 38 |
| **True negative** | 6006 | 5678 | 5315 | 3580 |
| **False positive** | 181 | 509 | 872 | 2607 |
| **False negative** | 13 | 4 | 2 | 0 |
| **Sensitivity (=Recall)** | 65.79% | 89.47% | 94.74% | 100.00% |
| **Specificity** | 97.07% | 91.77% | 85.91% | 57.86% |
| **Accuracy** | 96.88% | 91.76% | 85.96% | 58.12% |
| **Precision** | 12.14% | 6.26% | 3.96% | 1.44% |
| **F1-Score** | 20.49% | 11.70% | 7.61% | 2.83% |
|  |  |  |  |  |
| **Platypus 2** |  |  |  |  |
| Publications with invalid classification | 1 |  |  |  |
| **Threshold for classifier** | **5** | **4+** | **3+** | **2+** |
| **True positive** | 0 | 25 | 36 | 38 |
| **True negative** | 6187 | 5987 | 3865 | 20 |
| **False positive** | 0 | 200 | 2322 | 6167 |
| **False negative** | 38 | 13 | 2 | 0 |
| **Sensitivity (=Recall)** | 0.00% | 65.79% | 94.74% | 100.00% |
| **Specificity** | 100.00% | 96.77% | 62.47% | 0.32% |
| **Accuracy** | 99.39% | 96.58% | 62.67% | 0.93% |
| **Precision** | - | 11.11% | 1.53% | 0.61% |
| **F1-Score** | - | 19.01% | 3.01% | 1.22% |
|  |  |  |  |  |
| **Wolters_2018** |  | Total publications | 5019 |  |
| Relevant publications | 19 | Irrelevant publications | 5000 |  |
|  |  |  |  |  |
| **FlanT5** |  |  |  |  |
| Publications with invalid classification | 0 |  |  |  |
| **Threshold for classifier** | **5** | **4+** | **3+** | **2+** |
| **True positive** | 0 | 19 | 19 | 19 |
| **True negative** | 5019 | 3276 | 3275 | 19 |
| **False positive** | 0 | 1743 | 1744 | 5000 |
| **False negative** | 19 | 0 | 0 | 0 |
| **Sensitivity (=Recall)** | 0.00% | 100.00% | 100.00% | 100.00% |
| **Specificity** | 100.00% | 65.27% | 65.25% | 0.38% |
| **Accuracy** | 99.62% | 65.40% | 65.38% | 0.75% |
| **Precision** | - | 1.08% | 1.08% | 0.38% |
| **F1-Score** | - | 2.13% | 2.13% | 0.75% |
|  |  |  |  |  |
| **OHNC** |  |  |  |  |
| Publications with invalid classification | 2 |  |  |  |
| **Threshold for classifier** | **5** | **4+** | **3+** | **2+** |
| **True positive** | 0 | 18 | 19 | 19 |
| **True negative** | 5017 | 3786 | 1455 | 625 |
| **False positive** | 2 | 1233 | 3564 | 4394 |
| **False negative** | 19 | 1 | 0 | 0 |
| **Sensitivity (=Recall)** | 0.00% | 94.74% | 100.00% | 100.00% |
| **Specificity** | 99.96% | 75.43% | 28.99% | 12.45% |
| **Accuracy** | 99.58% | 75.51% | 29.26% | 12.78% |
| **Precision** | 0.00% | 1.44% | 0.53% | 0.43% |
| **F1-Score** | - | 2.83% | 1.05% | 0.86% |
|  |  |  |  |  |
| **Mixtral** |  |  |  |  |
| Publications with invalid classification | 0 |  |  |  |
| **Threshold for classifier** | **5** | **4+** | **3+** | **2+** |
| **True positive** | 11 | 19 | 19 | 19 |
| **True negative** | 4818 | 4582 | 4410 | 3339 |
| **False positive** | 201 | 437 | 609 | 1680 |
| **False negative** | 8 | 0 | 0 | 0 |
| **Sensitivity (=Recall)** | 57.89% | 100.00% | 100.00% | 100.00% |
| **Specificity** | 96.00% | 91.29% | 87.87% | 66.53% |
| **Accuracy** | 95.85% | 91.33% | 87.91% | 66.65% |
| **Precision** | 5.19% | 4.17% | 3.03% | 1.12% |
| **F1-Score** | 9.52% | 8.00% | 5.87% | 2.21% |
|  |  |  |  |  |
| **Platypus 2** |  |  |  |  |
| Publications with invalid classification | 0 |  |  |  |
| **Threshold for classifier** | **5** | **4+** | **3+** | **2+** |
| **True positive** | 0 | 17 | 19 | 19 |
| **True negative** | 5019 | 4756 | 2349 | 100 |
| **False positive** | 0 | 263 | 2670 | 4919 |
| **False negative** | 19 | 2 | 0 | 0 |
| **Sensitivity (=Recall)** | 0.00% | 89.47% | 100.00% | 100.00% |
| **Specificity** | 100.00% | 94.76% | 46.80% | 1.99% |
| **Accuracy** | 99.62% | 94.74% | 47.00% | 2.36% |
| **Precision** | - | 6.07% | 0.71% | 0.38% |
| **F1-Score** | - | 11.37% | 1.40% | 0.77% |

Table A1: Results of the LLM-based title-and-abstract screening depending on model and threshold of the classifiers on the ten published data sets.

| **CDSS_RO** |  | Total publications | 521 |  |
| --- | --- | --- | --- | --- |
| Relevant publications | 36 | Irrelevant publications | 485 |  |
|  |  |  |  |  |
| **FlanT5** |  |  |  |  |
| Publications with invalid classification | 0 |  |  |  |
| **Threshold for classifier** | **5** | **4+** | **3+** | **2+** |
| **True positive** | 0 | 36 | 36 | 36 |
| **True negative** | 485 | 61 | 61 | 0 |
| **False positive** | 0 | 424 | 424 | 485 |
| **False negative** | 36 | 0 | 0 | 0 |
| **Sensitivity (=Recall)** | 0.00% | 100.00% | 100.00% | 100.00% |
| **Specificity** | 100.00% | 12.58% | 12.58% | 0.00% |
| **Accuracy** | 93.09% | 18.62% | 18.62% | 6.91% |
| **Precision** | - | 7.83% | 7.83% | 6.91% |
| **F1-Score** | - | 14.52% | 14.52% | 12.93% |
|  |  |  |  |  |
| **OHNC** |  |  |  |  |
| Publications with invalid classification | 0 |  |  |  |
| **Threshold for classifier** | **5** | **4+** | **3+** | **2+** |
| **True positive** | 0 | 35 | 36 | 36 |
| **True negative** | 0 | 315 | 466 | 483 |
| **False positive** | 485 | 170 | 19 | 2 |
| **False negative** | 36 | 1 | 0 | 0 |
| **Sensitivity (=Recall)** | 0.00% | 97.22% | 100.00% | 100.00% |
| **Specificity** | 100.00% | 35.05% | 3.92% | 0.41% |
| **Accuracy** | 93.09% | 39.35% | 10.56% | 7.29% |
| **Precision** | - | 10.00% | 7.17% | 6.94% |
| **F1-Score** | - | 18.13% | 13.38% | 12.97% |
|  |  |  |  |  |
| **Mixtral** |  |  |  |  |
| Publications with invalid classification | 0 |  |  |  |
| **Threshold for classifier** | **5** | **4+** | **3+** | **2+** |
| **True positive** | 29 | 36 | 36 | 36 |
| **True negative** | 387 | 323 | 303 | 232 |
| **False positive** | 98 | 162 | 182 | 253 |
| **False negative** | 7 | 0 | 0 | 0 |
| **Sensitivity (=Recall)** | 80.56% | 100.00% | 100.00% | 100.00% |
| **Specificity** | 79.79% | 66.60% | 62.47% | 47.84% |
| **Accuracy** | 79.85% | 68.91% | 65.07% | 51.44% |
| **Precision** | 22.83% | 18.18% | 16.51% | 12.46% |
| **F1-Score** | 35.58% | 30.77% | 28.35% | 22.15% |
|  |  |  |  |  |
| **Platypus 2** |  |  |  |  |
| Publications with invalid classification | 0 |  |  |  |
| **Threshold for classifier** | **5** | **4+** | **3+** | **2+** |
| **True positive** | 0 | 33 | 36 | 36 |
| **True negative** | 485 | 366 | 120 | 0 |
| **False positive** | 0 | 119 | 365 | 485 |
| **False negative** | 36 | 3 | 0 | 0 |
| **Sensitivity (=Recall)** | 0.00% | 91.67% | 100.00% | 100.00% |
| **Specificity** | 100.00% | 75.46% | 24.74% | 0.00% |
| **Accuracy** | 93.09% | 76.58% | 29.94% | 6.91% |
| **Precision** | - | 21.71% | 8.98% | 6.91% |
| **F1-Score** | - | 35.11% | 16.48% | 12.93% |

Table A2: Results of the LLM-based title-and-abstract screening depending on model and threshold of the classifiers on the newly created data set.
