# Supplementary material for: Title and abstract screening for literature reviews using large language models: an exploratory study in the biomedical domain
Source: Syst Rev. 2024 Jun 15;13:158. doi: 10.1186/s13643-024-02575-4 (PMC11180407; doi:10.1186/s13643-024-02575-4)
Supplement: Supplementary file 4 — Supplementary Material 4: Appendix 4: Comparison with other approach. [file 13643_2024_2575_MOESM4_ESM.docx]

**Comparison of LLM-based title and abstract screening and approach of Natukunda et al.**

| **Appenzeller-Herzog_2020** | **LLM-based title and abstract screening (3+ classifiers)** | | | | **Natukunda et al.*** |
| --- | --- | --- | --- | --- | --- |
|  | **FlanT5** | **OHNC** | **Mixtral** | **Platypus 2** |  |
| **True positive** | 24 | 25 | 21 | 24 | 17 |
| **True negative** | 2078 | 973 | 2478 | 1681 | 2193 |
| **False positive** | 1375 | 2480 | 975 | 1772 | 1260 |
| **False negative** | 2 | 1 | 5 | 2 | 9 |
| **Invalid evaluations** | 0 | 0 | 0 | 0 | 0 |
| **Sensitivity (=Recall)** | 92.31% | 96.15% | 80.77% | 92.31% | 51.74% |
| **Specificity** | 60.18% | 28.18% | 71.76% | 48.68% | 57.89% |
| **Accuracy** | 60.42% | 28.69% | 71.83% | 48.83% | 63.52% |
| **Precision** | 1.72% | 1.00% | 2.11% | 0.67% | 1.33% |
| **F1-Score** | 3.37% | 1.98% | 4.11% | 1.34% | 2.61% |
|  |  |  |  |  |  |
| **Bos_2018** | **LLM-based title and abstract screening (3+ classifiers)** | | | | **Natukunda et al.** |
|  | **FlanT5** | **OHNC** | **Mixtral** | **Platypus 2** |  |
| **True positive** | 10 | 10 | 9 | 10 | 7 |
| **True negative** | 1399 | 1382 | 5561 | 2786 | 3151 |
| **False positive** | 4347 | 4364 | 185 | 2960 | 2595 |
| **False negative** | 0 | 0 | 1 | 0 | 3 |
| **Invalid evaluations** | 0 | 0 | 0 | 0 | 0 |
| **Sensitivity (=Recall)** | 100.00% | 100.00% | 90.00% | 100.00% | 70.00% |
| **Specificity** | 24.35% | 24.05% | 96.78% | 48.49% | 54.84% |
| **Accuracy** | 24.48% | 24.18% | 96.77% | 48.58% | 54.86% |
| **Precision** | 0.23% | 0.23% | 4.64% | 0.34% | 0.27% |
| **F1-Score** | 0.46% | 0.46% | 8.82% | 0.67% | 0.54% |
|  |  |  |  |  |  |
| **Donners_2020** | **LLM-based title and abstract screening (3+ classifiers)** | | | | **Natukunda et al.** |
|  | **FlanT5** | **OHNC** | **Mixtral** | **Platypus 2** |  |
| **True positive** | 14 | 15 | 14 | 15 | 12 |
| **True negative** | 107 | 18 | 174 | 63 | 284 |
| **False positive** | 538 | 627 | 471 | 582 | 359 |
| **False negative** | 1 | 0 | 1 | 0 | 3 |
| **Invalid evaluations** | 0 | 0 | 4 | 0 | 2 |
| **Sensitivity (=Recall)** | 93.33% | 100.00% | 93.33% | 100.00% | 80.00% |
| **Specificity** | 16.59% | 2.79% | 26.98% | 9.77% | 44.17% |
| **Accuracy** | 18.33% | 5.00% | 28.48% | 11.82% | 44.98% |
| **Precision** | 2.54% | 2.34% | 2.89% | 2.51% | 3.23% |
| **F1-Score** | 4.94% | 4.57% | 5.60% | 4.90% | 6.22% |
|  |  |  |  |  |  |
| **Jeyaraman_2021** | **LLM-based title and abstract screening (3+ classifiers)** | | | | **Natukunda et al.** |
|  | **FlanT5** | **OHNC** | **Mixtral** | **Platypus 2** |  |
| **True positive** | 85 | 96 | 23 | 96 | 1 |
| **True negative** | 290 | 17 | 1039 | 179 | 1096 |
| **False positive** | 808 | 1081 | 59 | 919 | 0 |
| **False negative** | 11 | 0 | 73 | 0 | 95 |
| **Invalid evaluations** | 0 | 0 | 0 | 0 | 2 |
| **Sensitivity (=Recall)** | 88.54% | 100.00% | 23.96% | 100.00% | 1.04% |
| **Specificity** | 26.41% | 1.55% | 94.63% | 16.30% | 100.00% |
| **Accuracy** | 31.41% | 9.46% | 88.94% | 23.03% | 92.03% |
| **Precision** | 9.52% | 8.16% | 28.05% | 9.46% | 100.00% |
| **F1-Score** | 17.19% | 15.08% | 25.84% | 17.28% | 2.06% |
|  |  |  |  |  |  |
| **Leenaars_2020** | **LLM-based title and abstract screening (3+ classifiers)** | | | | **Natukunda et al.** |
|  | **FlanT5** | **OHNC** | **Mixtral** | **Platypus 2** |  |
| **True positive** | 783 | 791 | 712 | 791 | 451 |
| **True negative** | 2942 | 1158 | 5685 | 2627 | 6188 |
| **False positive** | 5809 | 7593 | 3066 | 6124 | 2559 |
| **False negative** | 9 | 1 | 80 | 1 | 341 |
| **Invalid evaluations** | 0 | 0 | 13 | 0 | 4 |
| **Sensitivity (=Recall)** | 98.86% | 99.87% | 89.90% | 99.87% | 56.94% |
| **Specificity** | 33.62% | 13.23% | 64.96% | 30.02% | 70.74% |
| **Accuracy** | 39.03% | 20.42% | 67.03% | 35.82% | 69.60% |
| **Precision** | 11.88% | 9.43% | 18.85% | 11.44% | 14.98% |
| **F1-Score** | 21.21% | 17.24% | 31.16% | 20.53% | 23.72% |
|  |  |  |  |  |  |
| **Mejboom_2021** | **LLM-based title and abstract screening (3+ classifiers)** | | | | **Natukunda et al.** |
|  | **FlanT5** | **OHNC** | **Mixtral** | **Platypus 2** |  |
| **True positive** | 37 | 37 | 36 | 37 | 30 |
| **True negative** | 348 | 90 | 1324 | 842 | 376 |
| **False positive** | 1839 | 2097 | 863 | 1345 | 1809 |
| **False negative** | 0 | 0 | 1 | 0 | 7 |
| **Invalid evaluations** | 0 | 0 | 0 | 0 | 2 |
| **Sensitivity (=Recall)** | 100.00% | 100.00% | 97.30% | 100.00% | 81.08% |
| **Specificity** | 15.91% | 4.12% | 60.54% | 38.50% | 17.21% |
| **Accuracy** | 17.31% | 5.71% | 61.15% | 39.52% | 18.27% |
| **Precision** | 1.97% | 1.73% | 4.00% | 2.68% | 1.63% |
| **F1-Score** | 3.87% | 3.41% | 7.69% | 5.21% | 3.20% |
|  |  |  |  |  |  |
| **Muthu_2021** | **LLM-based title and abstract screening (3+ classifiers)** | | | | **Natukunda et al.** |
|  | **FlanT5** | **OHNC** | **Mixtral** | **Platypus 2** |  |
| **True positive** | 337 | 354 | 327 | 354 | 153 |
| **True negative** | 148 | 55 | 1456 | 348 | 1818 |
| **False positive** | 2752 | 2845 | 1444 | 2552 | 1082 |
| **False negative** | 17 | 0 | 27 | 0 | 201 |
| **Invalid evaluations** | 0 | 0 | 0 | 0 | 0 |
| **Sensitivity (=Recall)** | 95.20% | 100.00% | 92.37% | 100.00% | 43.22% |
| **Specificity** | 5.10% | 1.90% | 50.21% | 12.00% | 62.69% |
| **Accuracy** | 14.90% | 12.57% | 54.79% | 21.57% | 60.57% |
| **Precision** | 10.91% | 11.07% | 18.46% | 12.18% | 12.39% |
| **F1-Score** | 19.58% | 19.93% | 30.78% | 21.72% | 19.26% |
|  |  |  |  |  |  |
| **Oud_2018** | **LLM-based title and abstract screening (3+ classifiers)** | | | | **Natukunda et al.** |
|  | **FlanT5** | **OHNC** | **Mixtral** | **Platypus 2** |  |
| **True positive** | 20 | 20 | 20 | 20 | 19 |
| **True negative** | 469 | 304 | 796 | 412 | 381 |
| **False positive** | 564 | 729 | 237 | 621 | 652 |
| **False negative** | 0 | 0 | 0 | 0 | 1 |
| **Invalid evaluations** | 0 | 0 | 1 | 0 | 0 |
| **Sensitivity (=Recall)** | 100.00% | 100.00% | 100.00% | 100.00% | 95.00% |
| **Specificity** | 45.40% | 29.43% | 77.06% | 39.88% | 36.88% |
| **Accuracy** | 46.44% | 30.77% | 77.49% | 41.03% | 37.99% |
| **Precision** | 3.42% | 2.67% | 7.78% | 3.12% | 2.83% |
| **F1-Score** | 6.62% | 5.20% | 14.44% | 6.05% | 5.50% |
|  |  |  |  |  |  |
| **van_de_Schoot_**  **2018** | **LLM-based title and abstract screening (3+ classifiers)** | | | | **Natukunda et al.** |
|  | **FlanT5** | **OHNC** | **Mixtral** | **Platypus 2** |  |
| **True positive** | 36 | 35 | 36 | 36 | 19 |
| **True negative** | 1340 | 1258 | 5315 | 3865 | 2529 |
| **False positive** | 4847 | 4929 | 872 | 2322 | 3595 |
| **False negative** | 2 | 3 | 2 | 2 | 19 |
| **Invalid evaluations** | 0 | 0 | 0 | 1 | 63 |
| **Sensitivity (=Recall)** | 94.74% | 92.11% | 94.74% | 94.74% | 50.00% |
| **Specificity** | 21.66% | 20.33% | 85.91% | 62.47% | 41.30% |
| **Accuracy** | 22.10% | 20.77% | 85.96% | 62.67% | 41.35% |
| **Precision** | 0.74% | 0.71% | 3.96% | 1.53% | 0.53% |
| **F1-Score** | 1.46% | 1.40% | 7.61% | 3.01% | 1.04% |
|  |  |  |  |  |  |
| **Wolters_2018** | **LLM-based title and abstract screening (3+ classifiers)** | | | | **Natukunda et al.** |
|  | **FlanT5** | **OHNC** | **Mixtral** | **Platypus 2** |  |
| **True positive** | 19 | 19 | 19 | 19 | 19 |
| **True negative** | 3275 | 1455 | 4410 | 2349 | 3365 |
| **False positive** | 1744 | 3564 | 609 | 2670 | 1641 |
| **False negative** | 0 | 0 | 0 | 0 | 0 |
| **Invalid evaluations** | 0 | 2 | 0 | 0 | 13 |
| **Sensitivity (=Recall)** | 100.00% | 100.00% | 100.00% | 100.00% | 100.00% |
| **Specificity** | 65.25% | 28.99% | 87.87% | 46.80% | 67.22% |
| **Accuracy** | 65.38% | 29.26% | 87.91% | 47.00% | 67.34% |
| **Precision** | 1.08% | 0.53% | 3.03% | 0.71% | 1.14% |
| **F1-Score** | 2.13% | 1.05% | 5.87% | 1.40% | 2.26% |
|  |  |  |  |  |  |
| **CDSS_RO** | **LLM-based title and abstract screening (3+ classifiers)** | | | | **Natukunda et al.** |
|  | **FlanT5** | **OHNC** | **Mixtral** | **Platypus 2** |  |
| **True positive** | 36 | 36 | 36 | 36 | 34 |
| **True negative** | 61 | 466 | 303 | 120 | 192 |
| **False positive** | 424 | 19 | 182 | 365 | 293 |
| **False negative** | 0 | 0 | 0 | 0 | 2 |
| **Invalid evaluations** | 0 | 0 | 0 | 0 | 0 |
| **Sensitivity (=Recall)** | 100.00% | 100.00% | 100.00% | 100.00% | 94.44% |
| **Specificity** | 12.58% | 3.92% | 62.47% | 24.74% | 39.59% |
| **Accuracy** | 18.62% | 10.56% | 65.07% | 29.94% | 43.38% |
| **Precision** | 7.83% | 7.17% | 16.51% | 8.98% | 10.40% |
| **F1-Score** | 14.52% | 13.38% | 28.35% | 16.48% | 18.73% |
|  |  |  |  |  |  |

Table A3: Comparison of LLM-based title and abstract screening with 3+ classifiers and approach of Natukunda et al. *The data set Appenzeller-Herzog was also analyzed in the original publication of Natukunda et al. with reported sensitivity of 54.02% and a specificity of 67.03%. The values presented here are different as the list of keywords for this data set was newly created in the same way as it was created for the other data sets.
